# Optimizing Graphene Dispersion via Polymer Grafting

**DOI:** 10.1021/acs.macromol.4c02249

**Published:** 2025-01-02

**Authors:** Yang Wang, Wenjie Xia, Andrea Giuntoli

**Affiliations:** † Zernike Institute for Advanced Materials, University of Groningen, 9747 AG Groningen, The Netherlands; ‡ Department of Theoretical Physics & Center for Biophysics, Saarland University, 66123 Saarbrücken, Germany; § Department of Aerospace Engineering, Iowa State University, Ames, Iowa 50011, United States

## Abstract

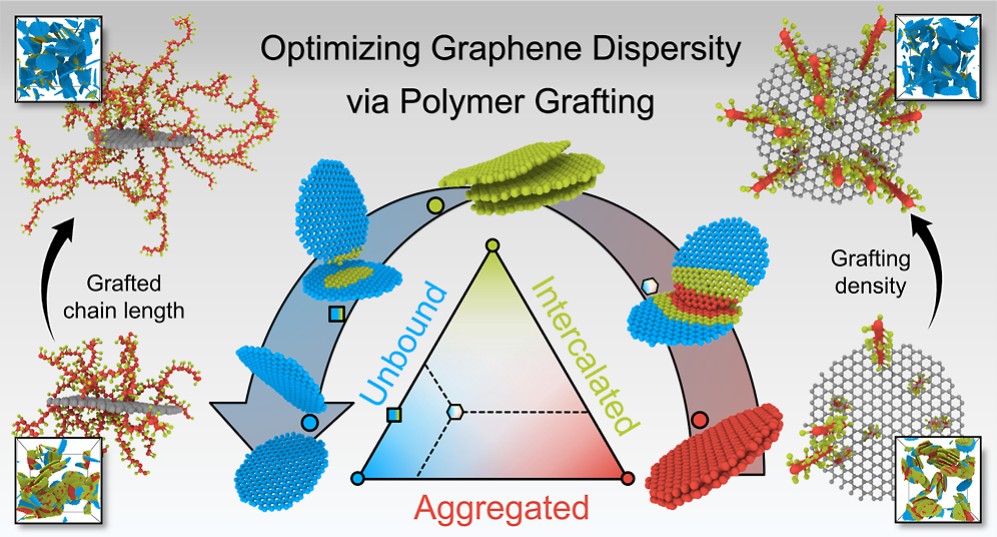

The dispersion of graphene sheets within a polymer matrix is critical for the performance of graphene-reinforced polymer nanocomposites, particularly in achieving optimal percolation and electrical conductivity. However, accurately characterizing and controlling the dispersion of 2D graphene in polymer melts remains a significant challenge due to the complex and varied configurations that graphene sheets can adopt. Herein, we employ coarse-grained molecular dynamics simulations to investigate how the grafting density (*g*) and grafted chain length (*n*) of poly(methyl methacrylate), p(MMA), affect graphene dispersion, where graphene is classified into three distinct morphologies, i.e., “aggregation”, “intercalated”, and “unbound”. We find that increasing *g* and *n* enhances graphene dispersion, evidenced by a higher dispersity parameter (*f*_d_), stronger interfacial interactions, greater Gaussian surface area of graphene clusters, and lower aggregation energy (*E*_Aggregation_). Our results also indicate that a higher *f*_d_ is linked to a higher Young’s modulus in the nanocomposite, reaching a maximum of 4.18 GPa. However, the electrical conductivity of nanocomposites initially rises with increasing *g* and *n* but declines beyond *g* > 5% and *n* > 10 due to reduced conductive pathways caused by graphene overdispersion, as revealed by the conductive edge analysis. Additionally, the free polymer fraction and chain length significantly influence toughness, and grafting p(MMA) chains on graphene slows down the dynamics of the surrounding polymer due to the intrinsic stiffness of graphene, an effect more pronounced at higher *f*_d_ (well dispersed). These findings present an effective approach for tuning and precisely characterizing graphene dispersity, clarifying its influence on material properties and forming the interfacial design of advanced nanocomposites reinforced with functional two-dimensional nanofillers.

## Introduction

Graphene, one-atom-thick two-dimensional layers of sp^2^-bonded carbon, has long been regarded as an ideal filler for improving the overall performance of graphene-based polymer nanocomposites due to its unique combination of high-surface area (2630 m^2^/g),^1^ thermal conductivity (5.30 ± 0.48 × 10^3^ W m^–1^ K^–1^),^2^ electrical conductivity (10^6^ S/m),^3^ and mechanical strength (the highest strength ≈130 GPa and Young’s modulus ≈1.0 TPa).^4^ The bulk availability of graphene has enabled graphene-reinforced polymer nanocomposites to be used in a wide range of industries,^5^ including aerospace,^6^ automotive,^7^ electronics,^8,9^ energy storage,^10,11^ and environmental engineering.^12^ In these applications, effectively dispersing graphene in a polymer matrix significantly enhances the mechanical, electrical, and thermal properties of the resulting composite material.^13^ However, the uniform mixing of polymer and graphene is challenging due to the chemical incompatibility between the two components, and the fact that the van der Waals forces and the high specific surface area of graphene potentially result in graphene agglomeration in the polymer matrix.^14,15^ Therefore, the graphene sheets are potentially aggregated experimentally, severely limiting further applications.^16^

A homogeneous dispersion of graphene within the polymer matrix is essential to unlock the full potential of graphene-enhanced materials.^17^ Hamidinejad et al.^18^ revealed that the thermal conductivity of graphene/high-density polyethylene nanocomposites dramatically increased from 2.09 ± 0.03 to 4.13 ± 0.12 W m^–1^ K^–1^ after in situ graphene exfoliation and dispersion under the same graphene volume fraction. Tang et al.^19^ discovered that the graphene/epoxy composites with well-dispersed thermally reduction graphite oxide (TRGO) exhibited a higher glass-transition temperature (*T*_g_) and significantly enhanced electrical conductivity compared to those with poorly dispersed RGO. Vasileiou et al.^20^ also proved that the better dispersion of TRGO resulted in a slight increase in the rheological and electrical percolation thresholds and a significant improvement in mechanical properties and thermal conductivity of TRGO-based linear low-density polyethylene composites. Therefore, various processing techniques have been implemented experimentally to achieve the homogeneous dispersion of graphene, including chemical functionalization of graphene,^21^ melt mixing,^22^ mechanically or ultrasonically exfoliating individual graphene sheets,^23,24^ and utilizing dispersing agents or compatibilizers.^25,26^ Among these methods, grafting polymer chains onto the edges or surfaces of graphene through surface engineering is a widely adopted technique to improve its dispersity in various media.^27^ Fang et al.^28^ demonstrated the ability to systematically tune the grafting density and chain length of polystyrene (PS) covalently bonded to single-layer graphene nanosheets. However, previous studies often used ambiguous terms such as “uniform” or “well-dispersed” without quantitatively describing the level of dispersion. Additionally, the diverse morphologies of graphene clusters in nanocomposites pose challenges in characterizing the dispersion and structure of graphene phases, which could significantly influence the mechanical and electrical properties of graphene–polymer nanocomposites.

Molecular dynamics (MD) simulation, especially the coarse-grained (CG) MD simulations, offers a promising avenue to complement experimental work and comprehensively address these challenges. Previous studies have extensively explored the dynamics,^29^ structure,^30,31^ dispersion,^32−34^ mechanical,^35^ viscoelastic,^36^ percolation^37^ of nanofiller reinforced polymer nanocomposites based on many design parameters, like graft and matrix polymer chemistry,^32^ shape, size, and aspect ratio of the fillers,^37,38^ grafted chain length,^39^ grafting density,^40^ polymer/nanofiller interaction strength,^36,41^ and so on. However, existing studies have predominantly focused on nanoparticles or nanorod fillers using a generic bead–spring CG model, which lacks chemistry specificity. In addition, accurate quantification of the dispersion state of two-dimensional materials like graphene has received significantly less theoretical attention as graphene can adopt diverse configurations within a polymer matrix such as face-to-face, edge-to-edge, and face-to-edge orientations.^42^ Konatham and Striolo^43^ investigated the dispersion state of ultrasmall graphene sheets (54 carbon atoms) grafted with short-branched alkanes using the united-atom model, revealing that this functionalization prevents graphene nanosheet agglomeration in organic solvents by leveraging excluded-volume effects. However, the dispersion state is characterized solely by the radial distribution function, lacking precise quantification of the graphene dispersion. By coupling the all-atom (AA) and coarse-grained (CG) models, Suter et al.^44^ systematically explored the aggregation of graphene and graphene oxide (GO) with varying degrees of oxidation in different polymer melts, providing invaluable insights into the behavior of graphene within polymer matrices at the molecular level. This work inspires us to understand the graphene dispersion mechanisms controlled by grafting, investigating for the first time both the spatial dispersion and electrical conductivity of graphene composites with a chemistry-specific coarse-grained model.

In this study, we systematically investigated and altered the molecular-level dispersion of graphene sheets in the polymer matrix by grafting the p(MMA) chain onto the graphene surface with different grafting densities (*g*) and grafted chain lengths (*n*) by using chemistry-specific graphene and p(MMA) CG models. By classifying the graphene sheet into three different morphologies, i.e., “aggregated”, “intercalated”, and “unbound”, we introduce the dispersity parameter *f*_d_ to characterize the graphene dispersion state. The impact of the graphene dispersion state on the mechanical, thermodynamic, structural, and electrical properties of the nanocomposite is reported. Results indicate that with increasing *g* and *n*, the dispersity of graphene improves, aggregation energy decreases, and the mechanical properties of the system are enhanced along with the slightly inhibited dynamics and nearly invariant glass-transition temperature. Additionally, a graph theory analysis uncovers that the electrical conductivity of the nanocomposites initially increases with increasing *g* and *n* but then declines when *g* > 5% and *n* > 10, which is caused by the overdispersion of graphene preventing sufficient conductive connections (number of edges) between graphene sheets. In general, this study mainly elucidates the intricate morphology of graphene within the polymer matrix and proposes design strategies for achieving optimal graphene dispersion. Moreover, our established modeling framework offers valuable insights into how the dispersion state of graphene influences the properties of graphene/polymer nanocomposites at the molecular level, allowing us to establish guidelines for advanced nanocomposite design.

## Methods

### Modeling Graphene/p(MMA) Nanocomposites

**Figure 1:**
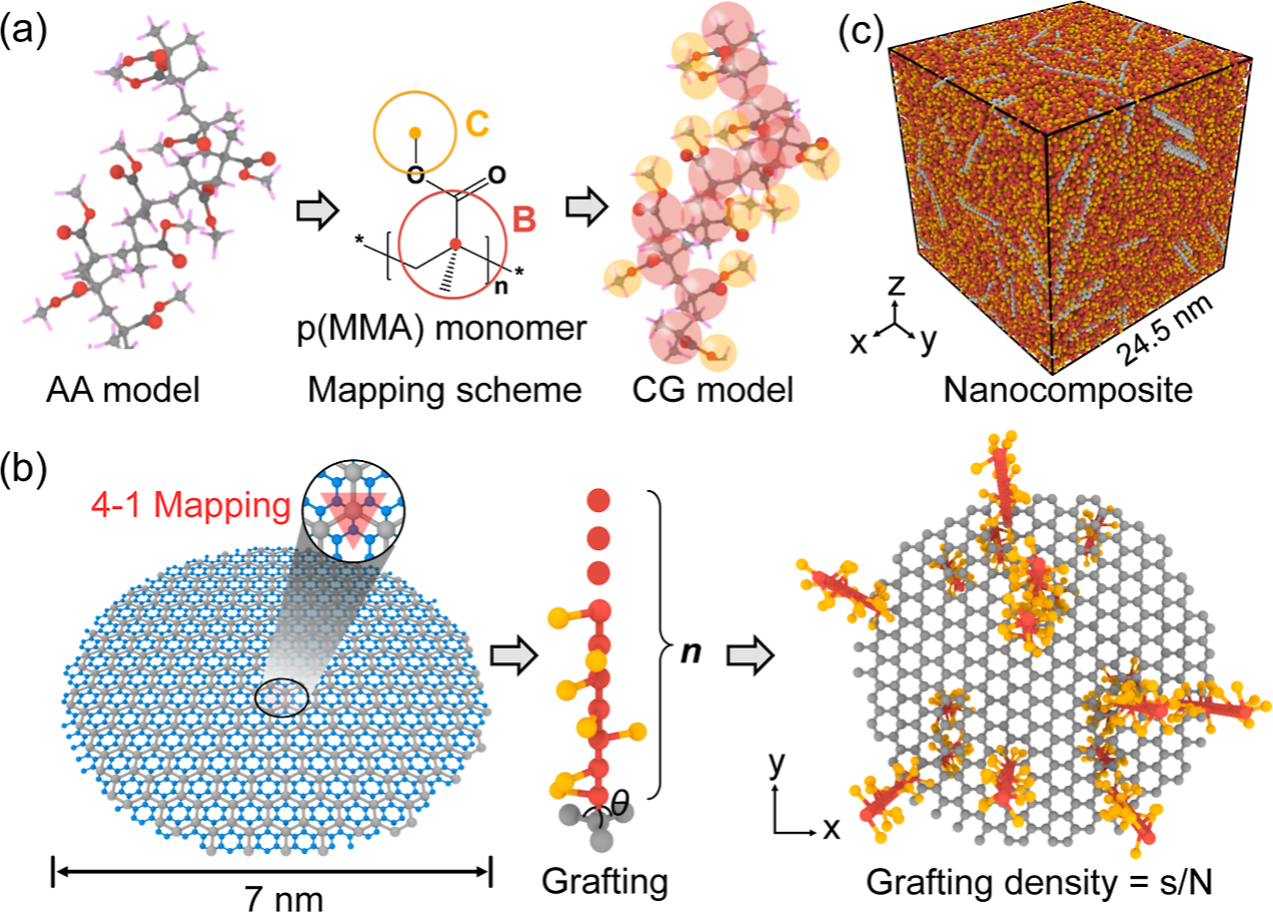
(a) Coarse-graining (left) all-atomistic (AA) poly(methyl methacrylate) p(MMA) model to the (right) coarse-grained (CG) model with the middle panel showing the chemical structure of p(MMA) and two-bead-per-monomer mapping scheme. (b) Fabrication of the fundamental p(MMA)-grafted graphene building block for the graphene/p(MMA) nanocomposite: (left panel) the CG model of graphene with each CG bead (gray) representing four carbon atoms (blue), i.e., 4–1 mapping scheme; (middle panel) the p(MMA) grafting process with *n* indicating the grafted polymer chain length, θ representing the angle between two bonded graphene beads and one polymer bead B; (right panel) the snapshot of the p(MMA)-grafted graphene building block with grafting density of *g* = *s*/*N*, where *s* and *N* stand for the number of grafting sites and the total number of graphene beads per graphene sheet, the p(MMA) chains are grafted on both sides of graphene with equal probability. (c) Snapshot of the bulk graphene/p(MMA) nanocomposite system with *g* = 5% and *n* = 30.

The atomistically informed poly(methyl methacrylate) p(MMA) CG model utilized in this work is derived from a study by Hsu et al.,^45^ which is based on a two-bead-*per*-monomer mapping scheme as depicted in Figure 1a. The backbone bead “B” represents the methacrylate group (C_4_O_2_H_5_), and the corresponding centers are located at the quaternary carbon atoms in the methacrylate group. The center of the side-chain bead “C” is located at the first side-chain carbon atom bound to the ester single-bonded oxygen. The CG graphene model is based on a 4-to-1 mapping scheme derived from the fine-grain all-atomistic model with the strain energy conservation approach,^46^ where four carbon atoms are grouped into one CG bead (type “A”), as shown in Figure 1b. The bonded and nonbonded parameters of the graphene and p(MMA) CG models are listed in Tables S1 and S2. The detailed parametrization of the p(MMA) and graphene CG models can be found in the previous work.^45,46^ Recently, we explored the crumpling behavior of single graphene sheets grafted with p(MMA) chains, including the conformational and mechanical properties.^47^ The key aspect in modeling the graphene/p(MMA) nanocomposite is the interfacial interaction between graphene and the polymer matrix, which determines the competitive adsorption of polymer chains to graphene versus graphene–graphene aggregation. In the present work, we calculated this interaction to be 0.29 and 0.33 J/m^2^ in well-dispersed and perfectly stacked layer nanocomposite systems (Figure S4e), which is comparable to the experimental work about the interfacial interaction between CNT and p(MMA).^48^ Additionally, extensive research utilizing graphene and p(MMA) CG models described above has been conducted to investigate the mechanical properties of graphene-reinforced p(MMA) nanocomposites.^49−52^ The range of grafted chains studied in our p(MMA)-grafted graphene nanocomposites can be synthesized by experiments, as proven by Vallés et al.,^53^ demonstrating the reliability of the p(MMA)-grafted graphene nanocomposites developed in this work. It is noted that despite the relatively small size of graphene (7 nm; Figure 1b) compared to experimental scales, we believe that analogous composites, in which both the graphene and the average size of polymers are proportionally scaled up, would exhibit similar properties. In addition, the radius of gyration of free p(MMA) chains in the graphene/p(MMA) nanocomposite system with *g* = 0.0% is about 18.8 Å, greatly smaller than the box size and demonstrates no significant size effect in this work, which is similar to the tensile deformation study of unentangled chain in various polymer thermoplastics, thermosets, and nanocomposite systems.^54−56^

**Figure 2:**
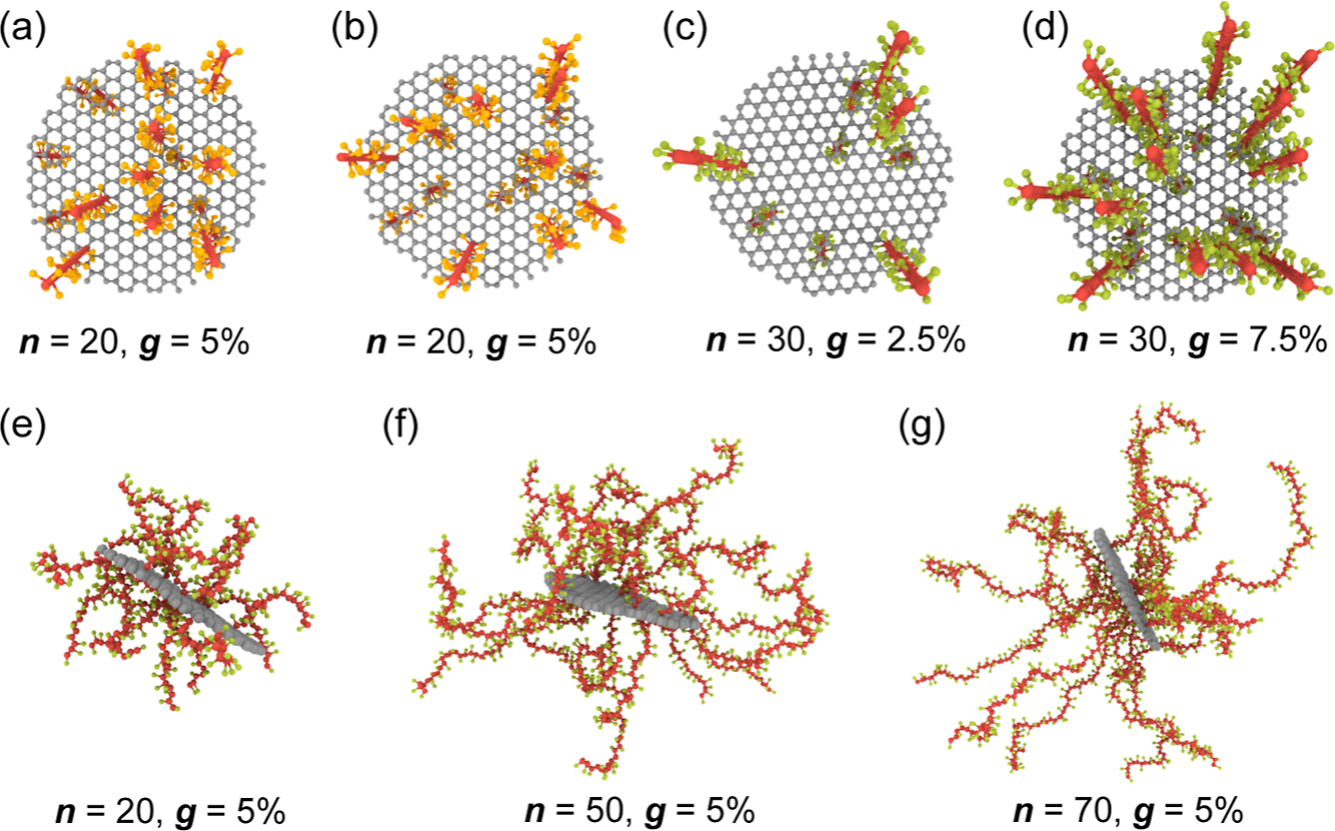
Different configurations of p(MMA)-grafted graphene nanoplatelets. (a,b) Representative snapshots of the initial p(MMA)-grafted graphene nanoplatelets with different grafting scenarios, where the grafting density is 5% and the grafted p(MMA) chain length is 20. (c,d) Representative snapshots of the p(MMA)-grafted graphene nanoplatelets with different grafting densities of 2.5% and 7.5% and a constant grafted chain length of 30. (e–g) Representative snapshots of the reaxed p(MMA)-grafted graphene nanoplatelets with different grafted p(MMA) chain lengths of 20, 50, and 70 and a constant grafting density of 5%.

To explore the effect of grafted chain length and grafting density on the graphene dispersion state, we built p(MMA)-grafted graphene nanoplatelets with varying grafted chain lengths and grafting densities of p(MMA) on the graphene surface, as shown in Figure 2. Grafting density is defined as *g* = *s*/*N*, where *s* and *N* represent the number of grafting sites and the total graphene beads of one graphene sheet, respectively, an intuitive representation to interpret the simulation results. For a more direct comparison with experimentally relevant quantities, we note that the number of grafted p(MMA) chains of graphene/p(MMA) nanocomposites are 0, 9, 18, 27 37, 46, and 55 per graphene sheet, and the grafting density can be described as 0, 0.23386, 0.46772, 0.70158, 0.96143, 1.19529, and 1.42915 chains/nm^2^ based on the radius of graphene (3.5 nm). In addition, as one graphene CG bead represents four carbon atoms (Figure 1b), the grafting density can be regarded as one p(MMA) chain per 151, 76, 50, 37, 30, and 25 carbon atoms, which is comparable to a similar experimental study in which the grafting density is about one p(MMA) chain per 40 carbon atoms.^53^ Then, we used an in-house Python script to achieve the grafting process (Figure 1b). Specifically, the grafting sites in each CG graphene sheet are randomly selected based on the grafting density, and then, p(MMA) chains with a certain grafted chain length (*n*) are chemically bonded to the grafting site on both sides of the graphene sheet (equal probability). Each graphene CG bead can be grafted with at most one polymer chain, and the distribution of grafting sites varies across each graphene sheet. Figure 2 shows representative snapshots of the p(MMA)-grafted graphene nanoplatelet with different grafting scenarios, grafted chain lengths, and grafting densities. The covalent bond between p(MMA) and graphene also follows the harmonic potential with the bond coefficient of 5 kcal/mol/Å^2^. We also add one angle type near the grafting site, i.e., θ angle in the middle panel of Figure 1b, using a harmonic angular potential with an angular stiffness constant of 5 kcal/mol/° and an equilibration angle value of 90°. It is revealed in the previous work that the nonbonded interaction contributes most during the uniaxial tensile deformation;^54,57^ therefore, we assume that the additional bond and angle near the grafting site would not influence the results of the current work due to their small amount and weaker coefficients. The detailed information on each simulation system is summarized in Table 1. A certain number of free p(MMA) chains with a chain length of 80 are randomly incorporated into the system to maintain a consistent total number of CG beads, ensuring a uniform graphene content of 8.5 wt % across all systems.

**Table 1:** Detailed Information of the p(MMA)-Grafted Graphene Building Block for Graphene/p(MMA) Nanocomposite Systems^a^

grafting density, g (%)	grafted chain length, n (mer)	free p(MMA) chain number	total number of beads
0.00	30	1194	209,540
2.50		1025	209,500
5.00		856	209,460
7.50		688	209,580
10.0		500	209,500
12.5		331	209,460
15.0		163	209,580
5.00	3	1160	209,500
	10	1081	209,460
	20	969	209,540
	40	744	209,540
	50	631	209,460
	60	519	209,540
	70	406	209,460

a The free p(MMA) chains with 80 monomers per chain are added into the box to ensure the same graphene content (8.5 wt %).

### Overview of the MD Simulation

The simulations in the present work are implemented in the Large-scale Atomic/Molecular Massively Parallel Simulator (LAMMPS) package (https://www.lammps.org/).^58^ The MDAnalysis (https://www.mdanalysis.org/) is used to analyze molecular dynamics trajectories.^59^ Visualizations are implemented using software OVITO.^60^ For each graphene/p(MMA) nanocomposite system, due to the different grafted chain lengths and grafting densities, a certain number of free p(MMA) chains with a chain length of 80 monomers are introduced to ensure the total bead counts of around 209,500, or the graphene content for each system is the same as 8.5 wt % (see Table 1). To build the graphene/p(MMA) nanocomposite system, 50 p(MMA)-grafted graphene building blocks and free p(MMA) chains with a chain length of 80 are randomly inserted into a large cubic box with a low initial density to avoid the free p(MMA) chain penetrating the graphene. The random distribution algorithm is based on the minimax space-filling design rule embedded in the Python package of *dopye* (https://github.com/tirthajyoti/doepy).

To begin the simulation, a randomly distributed initial velocity based on a temperature of 800 K is used to activate the structure. Then, the nanocomposite systems are energy minimized using the iterative conjugant gradient algorithm,^61^ and the system is gradually shrunk to a moderate density of 0.3 g/cm^3^ as the initial structure. It is noted that the graphene will not move significantly in the dense system due to its slow dynamics and steric obstacle by the surrounding polymers.^54^ Next, under the *NVT* ensemble, we utilized the modified cutoff distances (repulsive interaction) (Figure S1) to describe the interaction between p(MMA) chains in the nanocomposite system to accelerate the dynamics for 84 ns. With the pure repulsive interactions between p(MMA) chains, the graphene and polymer, graphene and graphene can still aggregate when they approach each other during the equilibration, hindered only by the steric interactions of the (grafted) polymer chains. This accelerates equilibration and prevents well-dispersed, out-of-equilibrium phases that contradict experimental findings,^62^ simply because the diffusion time of graphene sheets would be well beyond the time scales reached by MD simulations. The graphene–graphene and graphene-polymer attractive interactions on top of excluded-volume polymer–polymer interactions still allow for varying degrees of dispersion based on thermodynamics, as elaborated in Figure S1 of the Supporting Information. Finally, we perform 4 ns NPT annealing to condense the nanocomposite system with a pressure of 1 atm and temperature ramping from 800 to 300 K. After that, the system is further equilibrated under the *NPT* ensemble with a temperature of 300 K and a pressure of 1 atm for 4.4 ns, accompanied by sampling data with sampling intervals of 4 ps for the last 0.4 ns trajectory. The time step is 4 fs, and the periodic boundary conditions are applied in all directions to simulate the bulk behavior.

### Property Calculation

To characterize the chain dynamics, we calculate the mean square displacement (MSD), ⟨*r*^2^(*t*)⟩, of the CG beads
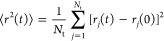
1where *r*_*j*_(*t*) is the position of the *j*th bead at time *t* and *N*_t_ is the total number of particles. The dynamical heterogeneity of the graphene/p(MMA) nanocomposite is characterized using the short-time fast dynamics property of the Debye–Waller factor (⟨*u*^2^⟩), which is determined as the MSD value at which *t* = 10 ps from a previous study.^63^ The virial stress was used to relate to the macroscopic (continuum) stress in molecular dynamics computations.
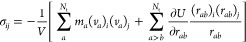
2where *V* is the system volume, *r*_*ab*_ stands for the distance between particles *a* and *b*, *U* represents the system’s total energy, and *m*_*a*_ and *v*_*a*_ are the mass and velocity of the particle, respectively. Specifically, the equation for the *i*, *j* components (where *i* and *j* = *x*, *y*, *z*) stands for the six-element symmetric stress tensor, and the tensile stress component is determined as σ_*xx*_. Three independent runs are performed in the *x*, *y*, and *z* directions for each system to improve the statistics.

## Results and Discussion

### Dispersion Analysis

**Figure 3:**
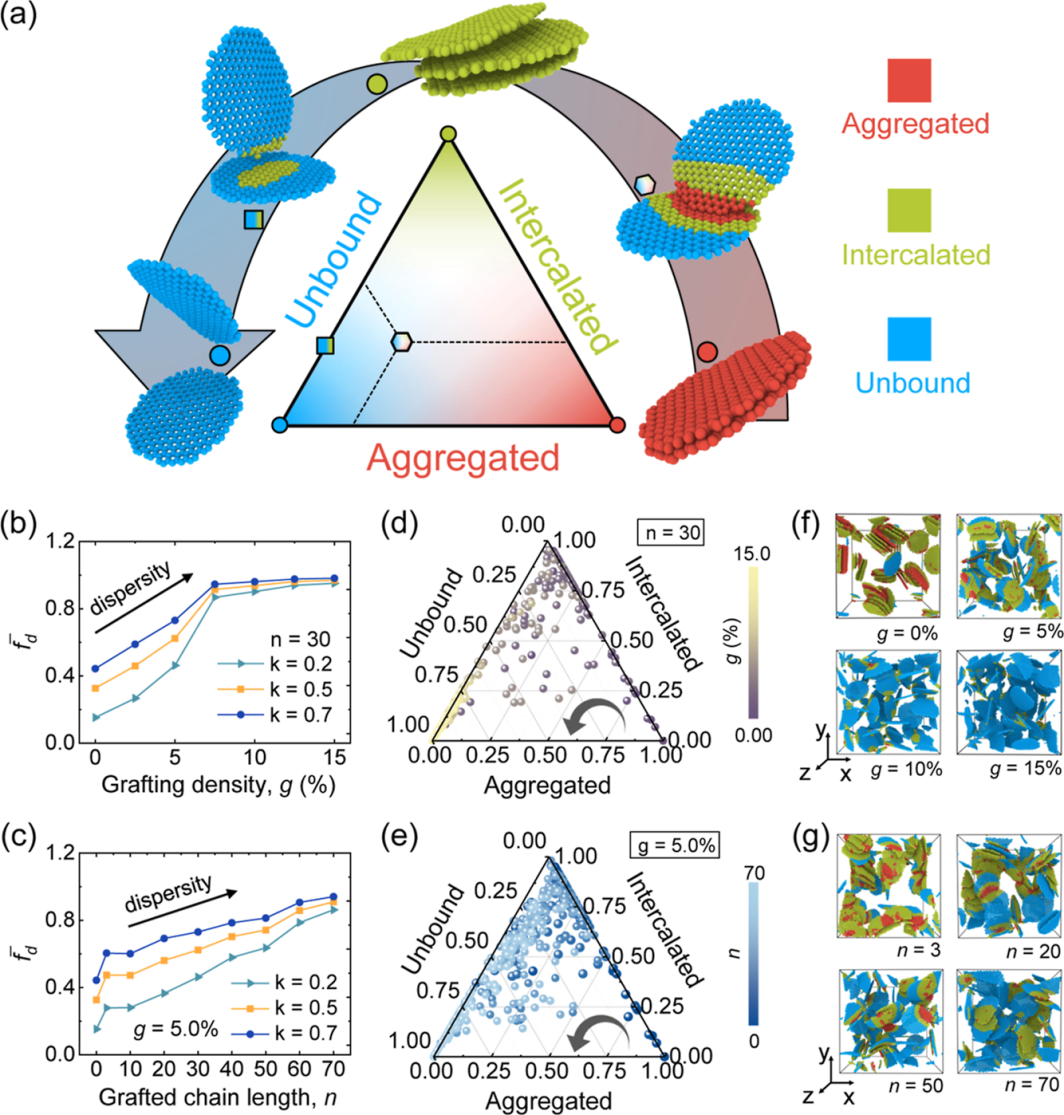
(a) Ternary phase diagram to illustrate the different distribution states of the graphene sheet. The CG beads in each graphene sheet are divided into aggregated (red), intercalated (green), and unbound (blue) states according to the distance between CG beads in different graphene sheets. Five representative distributions of graphene sheets in the ternary plot are depicted, signifying a gradual improvement in the dispersion state following the counterclockwise direction. The averaged  values in the equilibrium stage for the graphene/p(MMA) nanocomposite systems with (b) varying grafting densities *g* and (c) grafted chain lengths *n* under different *k* values. Ternary phase diagram of [*f*_A_, *f*_I_, *f*_U_] for each graphene sheet in the graphene/p(MMA) nanocomposites with different (d) grafting densities *g* and (e) grafted chain lengths *n*, with each point denoting one graphene sheet. Representative morphologies of the graphene sheet in the graphene/p(MMA) nanocomposites with (f) different grafting densities (i.e., *g* = 0.00%, 5.00%, 10.0%, and 15.0%) and (g) different grafted chain lengths (i.e., *n* = 3, 20, 50, and 70). The p(MMA) chains are omitted for clarity.

Experimentally, graphene sheets would easily get stacked or agglomerated in different modes during the fabrication due to their strong interlayer π–π interaction, such as the face-to-face, edge-to-edge, face-to-edge, or point-to-point modes, yielding insufficient contact between graphene and polymer matrix and further diminishing the performance of graphene-reinforced PNCs.^19^ One graphene flake in PNCs may experience varying environmental conditions simultaneously, for example, the combination of partial face-to-face and end-to-end. Therefore, we subdivided each graphene flake into three categories based on the particle nearest-neighbor distances (*d*) that for each graphene flake CG bead to beads in other graphene flakes:^44^ (i) *d* ≤ 8 Å, the particle is in an “aggregated” (A) environment (red beads in Figure 3a); (ii) 8 Å *d* ≤ 18 Å, the particle is in an “intercalated” (I) environment (green beads in Figure 3a); and (iii) *d* > 18 Å, the particle is in an “unbound” (U) environment (blue beads in Figure 3a). For each graphene flake, the numbers of particles in aggregated, intercalated, and unbound environments are *N*_A_, *N*_I_, and *N*_U_, respectively, and *N*_A_ + *N*_I_ + *N*_U_ = *N*, where *N* is the number of CG beads of one graphene flake, i.e., *N* = 370. Thus, we employ three parameters [*f*_A_, *f*_I_, *f*_U_] to describe the distribution state of CG beads in each graphene sheet, where *f*_A_ = *N*_A_/*N*, *f*_I_ = *N*_I_/*N*, *f*_U_ = *N*_U_/*N*, and *f*_A_ + *f*_I_ + *f*_U_ = 1. The overall dispersion state evolution of graphene flakes in the nanocomposite system versus simulation time is described by dispersity parameter *f*_d_(*t*) that combines the contributions of aggregated, intercalated, and unbound morphologies (eq 3)
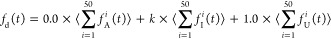
3where the coefficients for *f*_A_ and *f*_U_ are 0.0 and 1.0, respectively, denoting 0% and 100% contribute to the dispersity of graphene. As the contribution of intercalated graphene to dispersity lies between unbound and aggregated graphene, we specify a prefactor *k* to signify the extent to which the intercalated morphology impacts dispersity and ranges from 0 to 1. A larger *k* indicates a more significant contribution of the intercalated morphology to good dispersity. Coveney et al.^44^ introduced the method to categorize graphene morphologies based on nearest neighbor distances of CG sheet beads, and in the present work, we simultaneously combine the three morphologies to define a dispersity parameter of *f*_d_. Figure S2 represents the *f*_d_(*t*) variation as a function of simulation time *t*, and  is obtained in the final equilibration stage (last 0.4 ns). Generally,  represents that all graphene sheets are stacked together, and  represents that all graphene sheets are dispersed or in unbound morphology, a higher  indicates a nanocomposite system with good dispersion. Figure 3b,c presents that the equilibrated  of graphene/p(MMA) nanocomposites increases as rising values of *g* and *n* across different *k* values, indicating the improved dispersity of graphene (the black arrows in Figure 3b,c). It is noted that different *k* values lead to an overall shift in the  without changing the observed trends or affecting any of our observations or conclusions. In the rest of this article, results using *k* = 0.5 are reported. For graphene/p(MMA) nanocomposites exhibiting diverse grafting densities, a conspicuous transition point (*g* = 7.5%) is observed for different *k* values (Figure 3b), beyond which the augmentation of *g* exerts negligible influence on the dispersion as graphene sheets are far away from each other, as demonstrated by the radial distribution function in Figure S6 in the Supporting Information. Additionally, for the nanocomposites with different grafted chain lengths, short grafted PMMA chains (*n* = 3) onto graphene significantly improve dispersion (the drastic increase of *f*_d_ at *n* = 3), with further enhancement as the grafted chains lengthen (Figure 3c). Previous work also revealed that RGO with monomer-sized grafted functional groups could achieve better dispersity than pristine graphene.^44^

To explore the detailed morphologies of graphene within the polymer matrix, Figure 3d,e shows the [*f*_A_, *f*_I_, *f*_U_] data of graphene with different grafting densities and grafted chain lengths, where each data point represents one graphene sheet. It is noted that the data point [*f*_A_, *f*_I_, *f*_U_] denotes the fraction of aggregated, intercalated, and unbound graphene beads in each graphene, which is independent of *k*. The results suggest that with increasing *g* and *n*, the data points progressively converge counterclockwise toward the lower-left corner of the ternary phase diagram (Figure 3a), indicating that the proportion of graphene CG beads in the “unbound” morphology is increasing, leading to improved graphene dispersity within the system. Figure 3f,g displays representative snapshots of equilibrated nanocomposite systems with varying *g* and *n*, showing an increase in the proportion of unbound morphology (blue beads) as *g* and *n* rise. The distribution state of CG beads in each graphene sheet in the equilibrated state is summarized in Tables S3 and S4, where the [*f*_A_, *f*_I_, *f*_U_] = [1.0, 0.0, 0.0] is only detected in the graphene/p(MMA) nanocomposite with *g* = 0.0%, corresponding to the completely stacked red color graphene in Figure 3a.

The well-dispersed state of graphene enables substantial contact with p(MMA) and increases the contact area, both of which collectively impact the average interfacial interaction. Wan et al. also demonstrated that the interfacial interaction could serve as an indicator describing the degree of dispersion of GO in polymers.^64^ Herein, to quantitatively measure the graphene dispersion under different dispersion situations, we define the aggregation energy of the nanocomposite system, *E*_Aggregation_, as the interfacial energy between graphene and p(MMA), divided by the total Gaussian surface area of the graphene cluster.
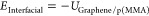
4
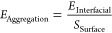
5where *U*_Graphene/p(MMA)_ denotes the nonbonded interaction (pair energy) between graphene and p(MMA). *S*_Surface_ denotes the Gaussian surface area, which generates an isosurface of a volumetric density field derived from the superposition of 3D Gaussian functions centered at each particle site.^65^Figure S4a,b illustrates the Gaussian surface derived from a single CG bead and graphene sheet. To quantify the effect of graphene dispersity on *E*_Aggregation_, we first create two graphene/p(MMA) laminated nanocomposites: one with perfectly dispersed graphene sheets and another with stacked graphene sheets (Figure S4c,d), where the *E*_Aggregation_ for the two graphene configurations is determined as 0.292 and 0.335 *J*/*m*^2^, respectively, neglecting the number of embedded graphene sheets and demonstrating the lower *E*_Aggregation_ value in the nanocomposite with good dispersity (Figure S4e). It is important to note that these two *E*_Aggregation_ values are not the upper and lower limits of aggregation energy as the graphene distribution is neither perfectly stacked nor fully dispersed within the nanocomposite system. Accordingly, within the framework of this base model, the present study posits that a higher *E*_Aggregation_ value denotes a system with reduced dispersity. In the previous work, Pramanik et al.^66^ introduced the concept of dispersion energy to quantify the dispersity of carbon nanotubes (CNTs) in various solvents and polymer solutions, defined as Δ*E*_d_ = −(*E*_dispersed_ – *E*_bundle_)/*S*_bundle_, where *E*_dispersed_ and *E*_bundle_ represent the total energy of the system at equilibrium for dispersed and bundle structures, respectively, corresponding to Figure S4c,d for our graphene/p(MMA) nanocomposites. Only the two surface areas of all graphene sheets in a stacked configuration can be effectively solvated. Therefore, Δ*E*_d_ is normalized to the Gaussian surface area of stacked graphene sheets rather than three separated graphene sheets. The calculated dispersion energy Δ*E*_d_ for graphene is 64.8 *mJ*/*m*^2^, which is larger than the dispersion energies of 20–40 *mJ*/*m*^2^ for CNTs in solution due to the two-dimensional geometry of graphene.^66^ Given the complex aggregation configurations of graphene within the polymer matrix compared to the one-dimensional CNT, we use *E*_Aggregation_ as an indicator to quantify the dispersion state of graphene in the current work. It is noted that the Gaussian surface area is also an indicator for quantifying the dispersion.

**Figure 4:**
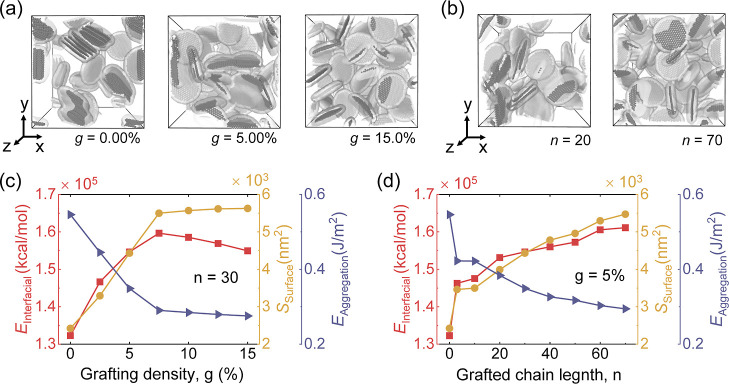
Demonstration of the Gaussian density surface area of the graphene clusters in the graphene/p(MMA) nanocomposites with different (a) grafting densities and (b) grafted chain lengths, where the p(MMA) chains are omitted for clarity. The variations of *E*_Interfacial_, *S*_Surface_, and *E*_Aggregation_ as a function of (c) grafting density and (d) grafted chain length of the graphene/p(MMA) nanocomposites.

Figure 4a,b depicts the Gaussian density surface distribution for the graphene clusters in graphene/p(MMA) nanocomposites with varying grafting densities and grafted chain lengths, and the corresponding *E*_Interfacial_ between graphene and p(MMA) and the *S*_Surface_ of graphene clusters is illustrated in Figure 4c,d. Results indicate that both *E*_Interfacial_ and *S*_Surface_ initially increase with higher grafting densities of the p(MMA) chain on graphene, while *S*_Surface_ plateaus and *E*_Interfacial_ exhibit a downward trend when *g* > 7.5% (Figure 4c), which is attributed to the insufficient contact between p(MMA) and graphene when grafting density exceeds 7.5% as elaborated by radial distribution function in Figure S6c in Supporting Information. As for the graphene/p(MMA) nanocomposites with varying grafted chain lengths, both *E*_Interfacial_ and *S*_Surface_ demonstrate a continuous increase with longer grafted p(MMA) chain lengths (Figure 4d). In both scenarios, the derived *E*_Aggregation_ decreases with increasing grafting density and grafted chain length, revealing an improved graphene dispersity. It is observed that there is a transition point in *E*_Aggregation_ when *g* > 7.5%, which corresponds with the variation of the average  in Figure 3b. The pronounced increase in *E*_Interfacial_ and *S*_Surface_, alongside the decline in *E*_Aggregation_ for nanocomposites from *n* = 0 to *n* = 3, aligns with the marked rise in  observed in Figure 3c.

### Thermomechanical and Dynamical Properties

Next, we investigated the thermomechanical properties of graphene/p(MMA) nanocomposites affected by *g* and *n*. Figure S8a illustrates density versus temperature curves for the graphene/p(MMA) nanocomposites, indicating a slight increase in density at each temperature with higher *g* and *n*. An increased in the *T*_g_ by approximately 14 K is observed in the nanocomposite compared to that of the pristine p(MMA) CG model (*T*_g_ = 385.2 ± 1.9 K), as depicted in Figure S8b. Vallés et al.^53^ reported a 15 °C increase in the *T*_g_ for graphene/p(MMA) nanocomposites with 5 wt % graphene loading and a grafting density of one p(MMA) chain per 40 carbon atoms. However, due to methodological uncertainties in determining *T*_g_,^67^ the *T*_g_ of graphene/p(MMA) nanocomposite shows no significant variation under the effect of different *g* and *n*, i.e., around 399 K, which is consistent with previous experiments on graphene/p(MMA) nanocomposites.^68^Figure S9a,b depicts the mean-square-displacement curves of graphene/p(MMA) nanocomposites, revealing a subtle decrease in dynamics with increasing *g* and *n*. We revealed in our recent work^54^ that graphene possesses higher molecular stiffness (lower mobility) than the polymer; thus, good dispersity of graphene (lower *f*_d_) would impede the mobility of adjacent polymer chains, leading to slower dynamics of nanocomposites. Fang et al.^28^ also found that the relaxation of polymer chains near the graphene surface was highly confined, particularly for segments near the surface. To further elucidate the differences in bead dynamics within the nanocomposite system, we examine the 3D molecular stiffness 1/⟨*u*^2^⟩ distribution in Figure S9c–f, where high values of 1/⟨*u*^2^⟩ are concentrated on the graphene sheet, indicating the rigid and high stiffness of graphene compared to p(MMA), consistent with findings from previous graphene/P3AT blending systems.^54^

**Figure 5:**
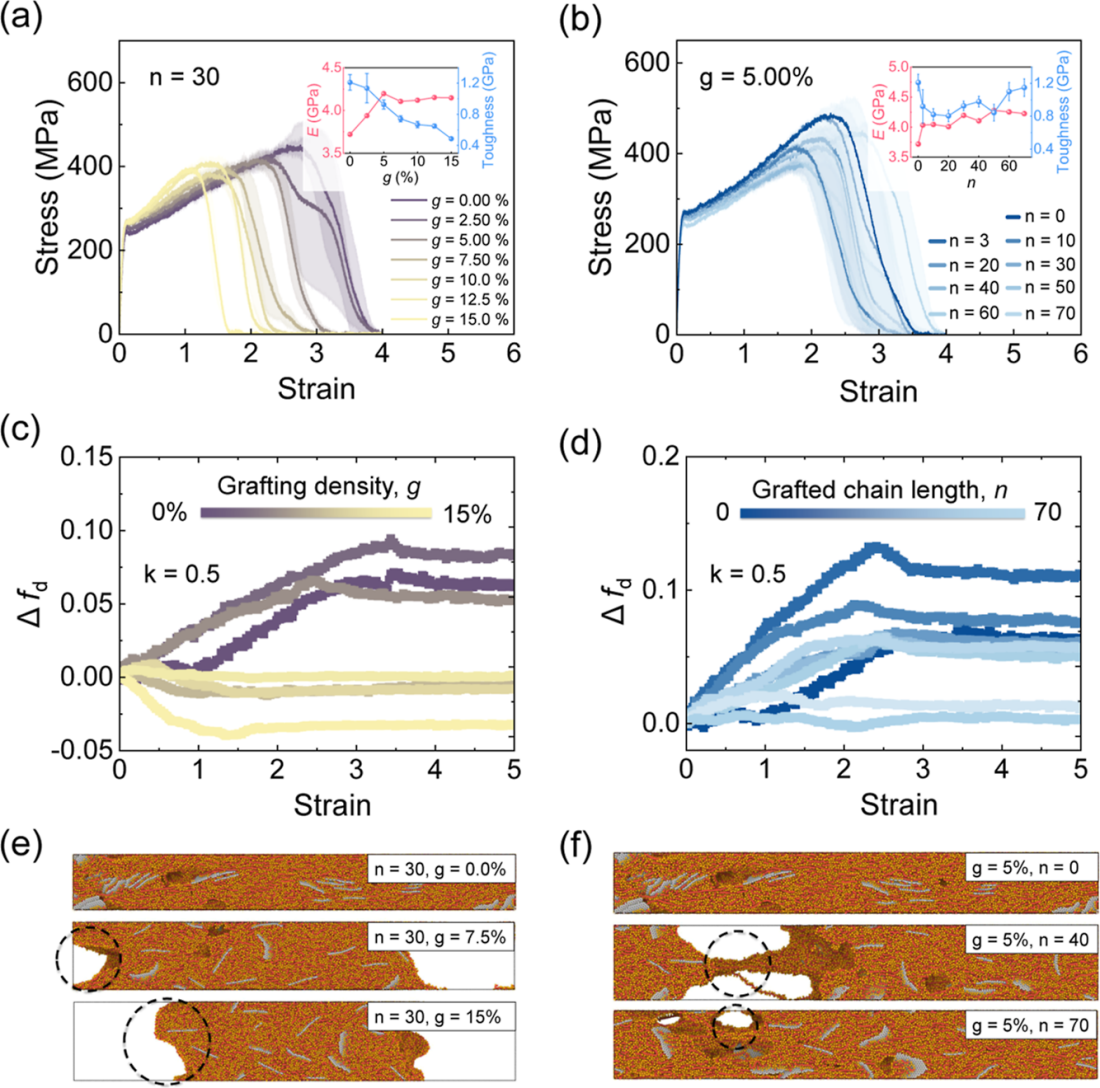
Stress–strain curves of graphene/p(MMA) nanocomposites with (a) different grafting densities *g* (*n* = 30) and (b) grafted chain lengths *n* (*g* = 5.00%). The error band is from the independent tensile test along the *x*, *y*, and *z* directions. The insets show Young’s modulus (*E*) and the toughness of the graphene/p(MMA) nanocomposites. The change of dispersity *f*_d_ (*k* = 0.5) as a function of strain for the graphene/p(MMA) nanocomposites (c) with fixed *n*, varying *g*, and (d) with fixed *g*, varying *n*. The snapshots of the nanocomposites with (e) different grafting densities and (f) grafted chain lengths at strain of 3.

Figure 5a,b shows the stress–strain curves of graphene/p(MMA) nanocomposites with different *g* and *n*, where Young’s modulus (*E*) and toughness are depicted in the insets. Results indicate that *E* increases with rising *g* but decreases slightly and then stabilizes when *g* > 5%. However, toughness decreases as grafting density increases, attributed to fewer free p(MMA) chains in a higher *g* system while maintaining a consistent graphene content (8.5 wt %) (Table 1). Previous studies have shown that polymer chain length plays a key role in determining the toughness of nanofiller-reinforced polymer nanocomposites and polymer thin films.^69^ Longer polymer chains enhance toughness by promoting sufficient chain sliding, increased interpenetration, and chain entanglement.^38,56^ In this study, the grafted p(MMA) chain length is fixed at 30 monomers per chain, and as *g* increases, the number of longer free p(MMA) chains (80 monomers per chain) decreases, leading to a corresponding decline in toughness (the inset is shown in Figure 5a). Figure 5e represents the snapshots of the graphene/p(MMA) nanocomposites at strain = 3. It is clear that for a constant *n*, the system with a larger *g* induces earlier fracture (the dotted circle in Figure 5e). In addition, for the system with *n* = 30 and *g* = 15%, all p(MMA)-grafted graphene building blocks are clustered at strain = 3 due to the few free p(MMA) chains (163 chains). For graphene/p(MMA) nanocomposites with varying *n*, Figure 5b reveals that Young’s modulus *E* generally increases as increasing *n*, with occasional decreases at specific lengths. However, the toughness decreases first and then increases with longer *n*, which is attributed to the combination effect of the grafted chain length and the fraction of free p(MMA) chains in the system. Specifically, at *n* = 0, the longer free p(MMA) chains primarily prevent material failure and enhance toughness. As *n* increases moderately, the free chains remain dominant but their proportion decreases due to the presence of grafted chains, resulting in reduced toughness. When the grafted chain length reaches *n* = 70, the system consists entirely of longer polymer chains (70 or 80 monomers), improving toughness. Figure 5f proves that the fracture of the system with *g* = 5% and *n* = 40 occurs earlier than that with *n* = 0 and *n* = 70 (the dotted circles are shown in the inset of Figure 5f). Overall, the fraction of isolated p(MMA) polymer chains, along with their respective lengths, significantly influences the mechanical properties of graphene/p(MMA) nanocomposites.

Next, we examined the evolution of the graphene dispersity *f*_d_ during stretching. As illustrated in Figure 5c,d, the nanocomposite system with a constant grafted chain length (*n* = 30) exhibits two distinct behaviors. In systems with lower grafting densities (*g* = 0%, 2.5%, and 5.0%), Δ*f*_d_ increases with larger strain values. Conversely, in systems with higher grafting densities (*g* = 7.5%, 10.0%, 12.5%, and 15%), Δ*f*_d_ decreases during stretching, albeit to a small extent. Figure 5e illustrates that at a high grafting density (*g* = 15%), the nanocomposite system contains few free p(MMA) chains, leading to the clustering of all building blocks upon stretching and resulting in no improvement in dispersion. For the system with a constant grafting density of *g* = 5% (Figure 5d), Δ*f*_d_ increases during stretching across various grafted chain lengths, indicating an enhanced dispersity of graphene upon stretching. Notably, systems with longer grafted chain lengths exhibit a smaller magnitude of increase in Δ*f*_d_ because their dispersity is already high before stretching (Figure 3c).

**Figure 6:**
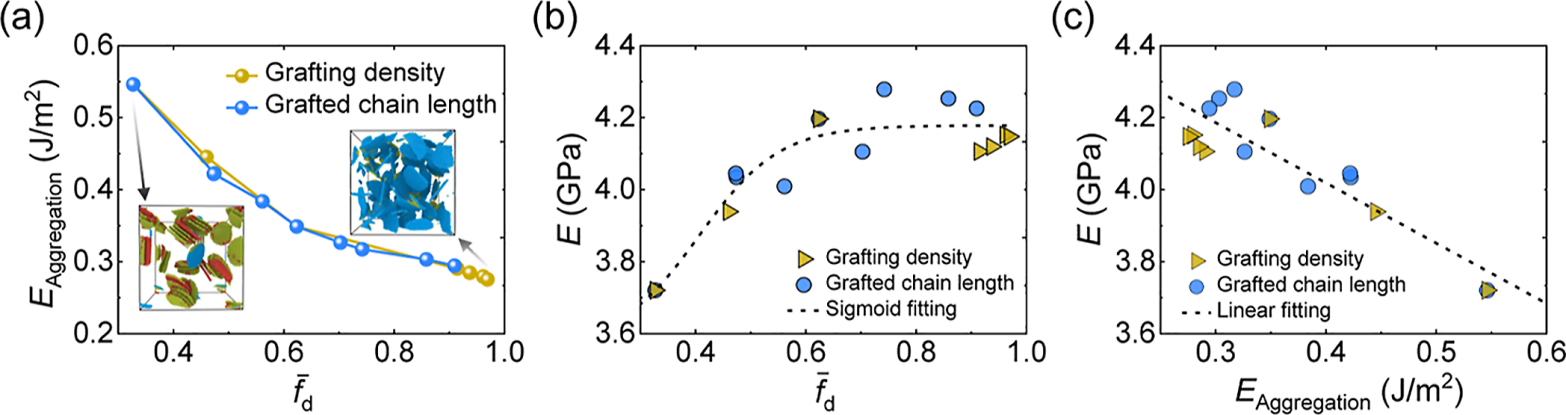
Aggregation energy *E*_Aggregation_ of graphene/p(MMA) nanocomposites as a function of the dispersity parameter  (where *k* = 0.5), showing that higher  (indicating improved dispersion) corresponds to lower *E*_Aggregation_. The insets depict graphene network snapshots for nanocomposites with grafting densities *g* = 0.0% and *g* = 15%. (b) Relationship between  and *E* across all systems, with the black dashed line indicating a sigmoidal fit and the plateau value at 4.18 GPa. (c) Linear correlation between *E*_Aggregation_ and *E*.

Furthermore, we investigate the relationship among , *E*_Aggregation_, and *E* in graphene/p(MMA) nanocomposites, revealing a nonlinear decrease of *E*_Aggregation_ as  rise across all nanocomposites with varying grafting densities and grafted chain lengths, as shown in Figure 6a. The results indicate that improved dispersity (higher ) of graphene correlates with a reduced *E*_Aggregation_. Gao et al.^70^ examined a composite of CNTs and GO as reinforcement for ordinary Portland cement (OPC), utilizing ultrasonication to optimize CNT and GO dispersion. The study demonstrated that a higher ultrasonication energy (time-controlled) markedly improves dispersion and the elastic modulus *E* initially, with *E* ultimately stabilizing at a plateau. Herein, we proceed to explore how *E* is influenced by  and *E*_Aggregation_ of the graphene/p(MMA) nanocomposite. Figure 6b illustrates a sigmoidal relationship between  and *E*, namely, , where *a*, *b*, *c*, and *d* are fitting parameters and the trend aligns with earlier findings.^70^ Specifically, *a* + *d* = 4.18 GPa denotes the stable maximum Young’s modulus and *c* = 0.41 represents the inflection point where the curve has the steepest slope. Additionally, Figure 6c indicates that higher *E*_Aggregation_ leads to a linear decrease in *E*, suggesting that a system with better dispersity (higher *f*_d_) exhibits a lower *E*_Aggregation_, resulting in an enhanced Young’s modulus.

### Percolation and Electrical Conductivity

Conductivity is a critical property of interest for developing 3D graphene-reinforced polymer nanocomposites for various functional applications. The quantum tunneling effect in graphene enables electrons to cross nanometer-scale barriers, significantly enhancing its potential in advanced electronic devices.^3,71^ Herein, we identify and preserve graphene clusters that span the entire simulation box in all three directions simultaneously using a tunneling distance of 2 nm,^72^ while discarding other sheets, as shown in Figure S10 in the Supporting Information. It is noted that the tunneling distance significantly affects the conductivity of nanocomposites as it governs the level of electron transfer at contact regions between adjacent nanosheets.^73^ Inspired by the seminal work of Klein and Randić,^74^ Ellens et al.^75^ proposed the effective graph resistance (also called Kirchhoff index), which is valuable in analyzing various network problems and can be written in terms of Laplacian eigenvalues. Based on the algebraic graph theory, we establish a network *G* with edges connecting the centers-of-mass of all graphene sheets in the percolated graphene cluster. Namely, *G* = (*V*, *E*), where *V* = {*v*_*i*_; *i* = 1, 2, ..., *k*} denotes the set of *k* nodes (center-of-mass of the graphene sheet, also called vertices), *E* = {(*v*_*i*_, *v*_*j*_); 1 ≤ *i* ≤ *k*, 1 ≤ *j* ≤ *k*} represents the set of edges (links between center-of-mass) that connects the *i*th and *j*th graphene sheets (center-of-mass) within percolation cluster; each edge is an unordered pair of nodes and treated as a resistor with unit resistance (1 Ω) according to Kirchhoff’s laws. The effective resistance between any two nodes, representing the total system resistance when a voltage is applied across them, can be determined appropriately by applying Kirchhoff’s voltage and current laws.^75^ For a given undirected graph, *G* = (*V*, *E*), the symmetric Laplacian matrix, also known as the Kirchhoff matrix, is defined as *L* = *D* – *A*. *D* and *A* are the degree matrix and adjacency matrix, respectively.^76^
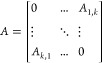
6
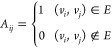
7where *k* denotes the number of graphene sheets in the percolated network within the simulation system, *A*_*ij*_ denotes an element of the matrix *A*, and each element *A*_*ij*_ corresponds to whether there is a direct connection (edge) between node *i* and node *j* (eq 7). Moreover, *A*_*ij*_ = 0 if *i* = *j*. The degree matrix (*D*) is a diagonal matrix, where *D*_*ii*_ represents the degree (number of connections) of node *i*, or the degree of a node is the number of edges connected to it, i.e., *D*_*ii*_ = *deg*(*i*) = ∑_*j*_*A*_*ij*_. Combining with the Moore-Penrose pseudoinverse of *L*, i.e., *L*^†^, we can determine the effective resistance *R*_*i*,*j*_ between nodes *v*_*i*_ and *v*_*j*_ as^74,75^
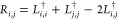
8

As the graphene network is highly anisotropic along three directions, we calculate the effective resistance (*R*_e_) using the Kirchhoff index (*K* = ∑_*i*<*j*_*R*_*ij*_) by looping all nodes in the network, i.e., *R*_e_ = *K* = ∑_*i*<*j*_*R*_*i*,*j*_. Then, the electrical conductivity can be obtained as σ_e_ = 1/*R*_e_.

**Figure 7:**
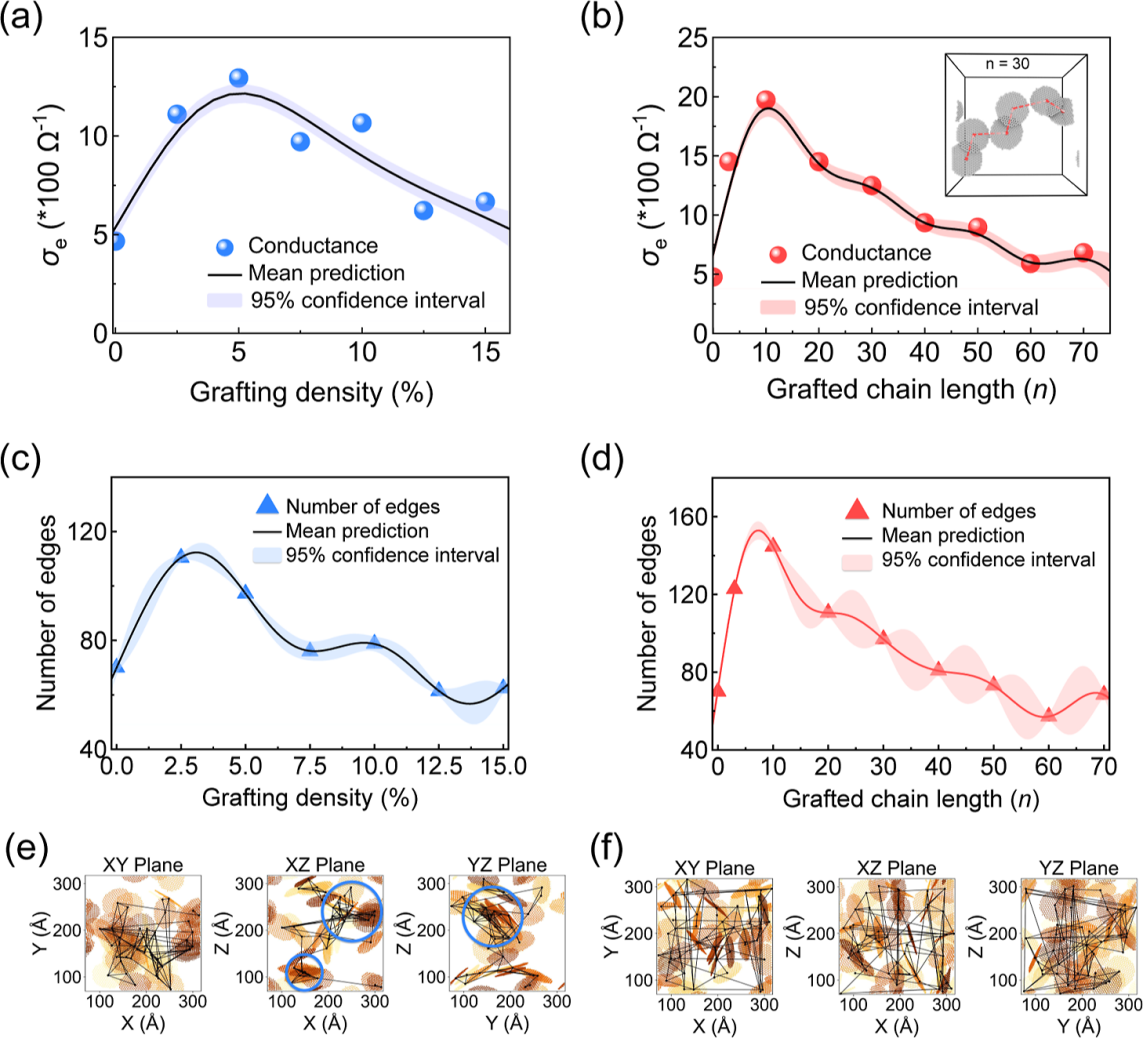
Electrical conductivity of graphene/p(MMA) nanocomposites with (a) different grafting densities and (b) grafted chain lengths, where the solid line and error band are the Gaussian progress regression prediction and the 95% confidence interval. The inset in panel (b) displays snapshots of one percolation path of nanocomposites with *n* = 30, with the red dashed line indicating the electronic transfer path. The number of edges in the graphene/p(MMA) nanocomposite systems with (c) different grafting densities and (d) grafted chain lengths. The error bar is smaller than the symbol. (e,f) Percolation network of graphene sheets within the graphene/p(MMA) nanocomposites with *g* = 2.5% and *g* = 5.0%, respectively. The blue circles indicate the aggregated edges.

Figure 7a,b shows that the optimal electrical conductivity in graphene/p(MMA) nanocomposites is achieved with a grafting density of *g* = 5% when fixing the grafting length *n* = 30, and with a grafting length of *n* = 10 when fixing the grafting density *g* = 5%. The electrical conductivity decreases when grafting density exceeds 5% and grafted chain length exceeds 10, which is attributed to the overdispersion of graphene. It is noted that the electrical conductivity is closely related to the number of edges in the system, as shown in Figure 7c,d. Herein, the impact of overdispersion on the electrical conductivity primarily refers to its effect on the number of conductive pathways within the system. While increasing the dispersity generally enhances mechanical properties, electrical conductivity is strongly dependent on graphene contact points and the structural integrity of the graphene network. The high graphene loading in the present work (8.5 wt %) is significantly higher than the reported electrical percolation threshold of graphene in the p(MMA) matrix of 2–4 wt %.^77^ Furthermore, Pan et al.^78^ demonstrated that although good CNT dispersion enhances interfacial adhesion between CNTs and the polymer, it also reduces the electrical conductivity of the composites, which occurs because the polymer matrix prevents sufficient contact between the nanofillers. Despite the limited number of simulation samples due to the large system size and time-consuming calculations, our results indicate that neither insufficient grafting nor dense grafting improves the electrical conductivity of the nanocomposites. Additionally, high grafting density and long grafted chains hinder conductivity by causing excessive polymer coverage on the graphene surface, i.e., overdispersion. Achieving optimal conductivity requires a careful balance of grafting density and chain length, highlighting the critical importance of these factors in optimizing the conductive properties of graphene-based nanocomposites.

## Conclusions

Based on chemistry-specific coarse-grained models and MD simulations, this study investigated the polymer-grafted graphene dispersion within a polymer matrix of graphene/p(MMA) nanocomposites at molecular resolution, considering varying grafting densities and grafted chain lengths. We characterized the dispersion state of graphene through a dispersity parameter, *f*_d_, considering three different morphologies of graphene sheets: “aggregated”, “intercalated”, and “unbound”. Results revealed that the repulsive interaction of the polymer matrix can significantly influence the dispersion state of pristine graphene, which should be carefully considered in collaboration with experimental work. Increasing the grafting density and grafted chain length improves the dispersion state of graphene (higher *f*_d_ value), resulting in lower aggregation energy, higher interfacial interaction energy, larger Gaussian surface area, and enhanced mechanical property (Young’s modulus). It is noted that the toughness is highly related to the chain length of the polymer and the fraction of free polymer in the system. The good dispersity of graphene results in slightly slower dynamics due to the significantly higher molecular stiffness of the graphene compared to the polymer, which also leads to a higher *T*_g_ value for the nanocomposite compared to the pristine p(MMA) system. However, *T*_g_ is not sensitive across different grafting densities and grafted chain lengths. Finally, using both graph theory and transport modeling, we demonstrate that the electrical conductivity of graphene/p(MMA) nanocomposites initially increases with higher grafting density and grafted chain length due to the formation of more conductive edges within the system. However, when the grafting density surpasses 5.0% and the grafted chain length exceeds 10, the conductivity decreases, which is attributed to the overdispersion of graphene by the surrounding polymer chains, leading to reduced contact between them. These coarse-grained molecular simulations significantly enhance our understanding of graphene aggregation and dispersion, providing valuable insights into optimizing the graphene distribution or assembly within the polymer matrix using a grafting approach. Our findings not only shed light on the adjustable properties influenced by graphene dispersion but also present significant potential for developing advanced nanocomposites incorporating additional two-dimensional nanofillers. Furthermore, factors such as the interaction between graphene and polymers,^79^ polymer matrix properties, graphene morphology (flake sizes and shapes), grafting scenarios, and temperature can greatly affect graphene dispersion and merit further investigation.

## Data Availability

To promote Open Science practices, the LAMMPS code used to perform our simulations is available on the GitHub page of our group: https://github.com/giuntoli-group.

## Supporting Information Available

Details of the CG models; simulation protocol; dispersion analysis; structure property; thermal, dynamic, and mechanical properties; percolation and conductivity; and CG bonded and nonbonded potential (PDF)

## References

[ref1] Stoller M. D.; Park S.; Zhu Y.; An J.; Ruoff R. S. Graphene-based ultracapacitors. Nano Lett. 2008, 8, 3498–3502. 10.1021/nl802558y. 18788793

[ref2] Balandin A. A.; Ghosh S.; Bao W.; Calizo I.; Teweldebrhan D.; Miao F.; Lau C. N. Superior thermal conductivity of single-layer graphene. Nano Lett. 2008, 8, 902–907. 10.1021/nl0731872. 18284217

[ref3] Zhuang Y.; Zheng K.; Cao X.; Fan Q.; Ye G.; Lu J.; Zhang J.; Ma Y. Flexible graphene nanocomposites with simultaneous highly anisotropic thermal and electrical conductivities prepared by engineered graphene with flat morphology. ACS Nano 2020, 14, 11733–11742. 10.1021/acsnano.0c04456. 32865991

[ref4] Lee C.; Wei X.; Kysar J. W.; Hone J. Measurement of the elastic properties and intrinsic strength of monolayer graphene. Science 2008, 321, 385–388. 10.1126/science.1157996. 18635798

[ref5] Stanford M. G.; Bets K. V.; Luong D. X.; Advincula P. A.; Chen W.; Li J. T.; Wang Z.; McHugh E. A.; Algozeeb W. A.; Yakobson B. I.; et al. Flash Graphene Morphologies. ACS Nano 2020, 14, 13691–13699. 10.1021/acsnano.0c05900. 32909736

[ref6] Das P.; Banerjee S.; Das N. C. Polymer Nanocomposites Containing Graphene; Elsevier, 2022; pp 683–711.

[ref7] Shah V.; Bhaliya J.; Patel G. M.; Deshmukh K. Advances in polymeric nanocomposites for automotive applications: A review. Polym. Adv. Technol. 2022, 33, 3023–3048. 10.1002/pat.5771.

[ref8] You R.; Liu Y.-Q.; Hao Y.-L.; Han D.-D.; Zhang Y.-L.; You Z. Laser fabrication of graphene-based flexible electronics. Adv. Mater. 2020, 32, 1901981 10.1002/adma.201901981. 31441164

[ref9] Pan K.; Fan Y.; Leng T.; Li J.; Xin Z.; Zhang J.; Hao L.; Gallop J.; Novoselov K. S.; Hu Z. Sustainable production of highly conductive multilayer graphene ink for wireless connectivity and IoT applications. Nat. Commun. 2018, 9, 5197 10.1038/s41467-018-07632-w. 30518870 PMC6281590

[ref10] Chen Z.; An X.; Dai L.; Xu Y. Holey graphene-based nanocomposites for efficient electrochemical energy storage. Nano Energy 2020, 73, 104762 10.1016/j.nanoen.2020.104762.

[ref11] Wang M.; Duan X.; Xu Y.; Duan X. Functional three-dimensional graphene/polymer composites. ACS Nano 2016, 10, 7231–7247. 10.1021/acsnano.6b03349. 27403991

[ref12] Karthik V.; Selvakumar P.; Senthil Kumar P.; Vo D.-V. N.; Gokulakrishnan M.; Keerthana P.; Tamil Elakkiya V.; Rajeswari R. Graphene-based materials for environmental applications: a review. Environ. Chem. Lett. 2021, 19, 3631–3644. 10.1007/s10311-021-01262-3.

[ref13] Kinloch I. A.; Suhr J.; Lou J.; Young R. J.; Ajayan P. M. Composites with carbon nanotubes and graphene: An outlook. Science 2018, 362, 547–553. 10.1126/science.aat7439. 30385571

[ref14] Zhao X.; Zhang Q.; Chen D.; Lu P. Enhanced mechanical properties of graphene-based poly (vinyl alcohol) composites. Macromolecules 2010, 43, 2357–2363. 10.1021/ma902862u.

[ref15] Huang H.; Shi H.; Das P.; Qin J.; Li Y.; Wang X.; Su F.; Wen P.; Li S.; Lu P.; et al. The Chemistry and Promising Applications of Graphene and Porous Graphene Materials. Adv. Funct. Mater. 2020, 30, 1909035 10.1002/adfm.201909035.

[ref16] Ghanem A. F.; Youssef A. M.; Abdel Rehim M. H. Hydrophobically modified graphene oxide as a barrier and antibacterial agent for polystyrene packaging. J. Mater. Sci. 2020, 55, 4685–4700. 10.1007/s10853-019-04333-7.

[ref17] Stankovich S.; Dikin D. A.; Dommett G. H.; Kohlhaas K. M.; Zimney E. J.; Stach E. A.; Piner R. D.; Nguyen S. T.; Ruoff R. S. Graphene-based composite materials. Nature 2006, 442, 282–286. 10.1038/nature04969. 16855586

[ref18] Hamidinejad S. M.; Chu R. K.; Zhao B.; Park C. B.; Filleter T. Enhanced thermal conductivity of graphene nanoplatelet–polymer nanocomposites fabricated via supercritical fluid-assisted in situ exfoliation. ACS Appl. Mater. Interfaces 2018, 10, 1225–1236. 10.1021/acsami.7b15170. 29226667

[ref19] Tang L.-C.; Wan Y.-J.; Yan D.; Pei Y.-B.; Zhao L.; Li Y.-B.; Wu L.-B.; Jiang J.-X.; Lai G.-Q. The effect of graphene dispersion on the mechanical properties of graphene/epoxy composites. Carbon 2013, 60, 16–27. 10.1016/j.carbon.2013.03.050.

[ref20] Vasileiou A. A.; Kontopoulou M.; Docoslis A. A noncovalent compatibilization approach to improve the filler dispersion and properties of polyethylene/graphene composites. ACS Appl. Mater. Interfaces 2014, 6, 1916–1925. 10.1021/am404979g. 24422418

[ref21] Kuila T.; Bose S.; Mishra A. K.; Khanra P.; Kim N. H.; Lee J. H. Chemical functionalization of graphene and its applications. Prog. Mater. Sci. 2012, 57, 1061–1105. 10.1016/j.pmatsci.2012.03.002.

[ref22] Allahbakhsh A. PVC/rice straw/SDBS-modified graphene oxide sustainable Nanocomposites: Melt mixing process and electrical insulation characteristics. Composites, Part A 2020, 134, 105902 10.1016/j.compositesa.2020.105902.

[ref23] Li Z.; Young R. J.; Backes C.; Zhao W.; Zhang X.; Zhukov A. A.; Tillotson E.; Conlan A. P.; Ding F.; Haigh S. J.; et al. Mechanisms of Liquid-Phase Exfoliation for the Production of Graphene. ACS Nano 2020, 14, 10976–10985. 10.1021/acsnano.0c03916. 32598132

[ref24] Tyurnina A. V.; Tzanakis I.; Morton J.; Mi J.; Porfyrakis K.; Maciejewska B. M.; Grobert N.; Eskin D. G. Ultrasonic exfoliation of graphene in water: A key parameter study. Carbon 2020, 168, 737–747. 10.1016/j.carbon.2020.06.029.

[ref25] Ho Q. B.; Kontopoulou M. Compatibilized polypropylene nanocomposites containing expanded graphite and graphene nanoplatelets. Polym. Eng. Sci. 2021, 61, 1116–1128. 10.1002/pen.25647.

[ref26] Wang B.; Ye X.; Wang B.; Li X.; Xiao S.; Liu H. Reactive graphene as highly efficient compatibilizer for cocontinuous poly (lactic acid)/poly (ε-caprolactone) blends toward robust biodegradable nanocomposites. Compos. Sci. Technol. 2022, 221, 109326 10.1016/j.compscitech.2022.109326.

[ref27] Eskandari P.; Abousalman-Rezvani Z.; Roghani-Mamaqani H.; Salami-Kalajahi M.; Mardani H. Polymer grafting on graphene layers by controlled radical polymerization. Adv. Colloid Interface Sci. 2019, 273, 102021 10.1016/j.cis.2019.102021. 31473461

[ref28] Fang M.; Wang K.; Lu H.; Yang Y.; Nutt S. Single-layer graphene nanosheets with controlled grafting of polymer chains. J. Mater. Chem. 2010, 20, 1982–1992. 10.1039/b919078c.

[ref29] Zhu Y.; Giuntoli A.; Zhang W.; Lin Z.; Keten S.; Starr F. W.; Douglas J. F. The effect of nanoparticle softness on the interfacial dynamics of a model polymer nanocomposite. J. Chem. Phys. 2022, 157, 094901 10.1063/5.0101551. 36075703

[ref30] Midya J.; Rubinstein M.; Kumar S. K.; Nikoubashman A. Structure of polymer-grafted nanoparticle melts. ACS Nano 2020, 14, 15505–15516. 10.1021/acsnano.0c06134. 33084300 PMC8056455

[ref31] Chan S. Y.; Jhalaria M.; Huang Y.; Li R.; Benicewicz B. C.; Durning C. J.; Vo T.; Kumar S. K. Local Structure of Polymer-Grafted Nanoparticle Melts. ACS Nano 2022, 16, 10404–10411. 10.1021/acsnano.2c00643. 35816726

[ref32] Jayaraman A. Polymer grafted nanoparticles: Effect of chemical and physical heterogeneity in polymer grafts on particle assembly and dispersion. J. Polym. Sci., Part B: Polym. Phys. 2013, 51, 524–534. 10.1002/polb.23260.

[ref33] Shen J.; Li X.; Shen X.; Liu J. Insight into the dispersion mechanism of polymer-grafted nanorods in polymer nanocomposites: A molecular dynamics simulation study. Macromolecules 2017, 50, 687–699. 10.1021/acs.macromol.6b02284.

[ref34] Koh C.; Grest G. S.; Kumar S. K. Assembly of polymer-grafted nanoparticles in polymer matrices. ACS Nano 2020, 14, 13491–13499. 10.1021/acsnano.0c05495. 33030334

[ref35] Midya J.; Cang Y.; Egorov S. A.; Matyjaszewski K.; Bockstaller M. R.; Nikoubashman A.; Fytas G. Disentangling the role of chain conformation on the mechanics of polymer tethered particle materials. Nano Lett. 2019, 19, 2715–2722. 10.1021/acs.nanolett.9b00817. 30913883 PMC6463242

[ref36] Liu J.; Zhang L.; Cao D.; Wang W. Static, rheological and mechanical properties of polymer nanocomposites studied by computer modeling and simulation. Phys. Chem. Chem. Phys. 2009, 11, 11365–11384. 10.1039/b913511a. 20024405

[ref37] Lu S.; Wu Z.; Jayaraman A. Molecular modeling and simulation of polymer nanocomposites with nanorod fillers. J. Phys. Chem. B 2021, 125, 2435–2449. 10.1021/acs.jpcb.1c00097. 33646794

[ref38] Hansoge N. K.; Huang T.; Sinko R.; Xia W.; Chen W.; Keten S. Materials by design for stiff and tough hairy nanoparticle assemblies. ACS Nano 2018, 12, 7946–7958. 10.1021/acsnano.8b02454. 29975847

[ref39] Dahal U.; Dormidontova E. E. Chain conformation and hydration of polyethylene oxide grafted to gold nanoparticles: curvature and chain length effect. Macromolecules 2020, 53, 8160–8170. 10.1021/acs.macromol.0c01499.

[ref40] Shen J.; Liu J.; Gao Y.; Cao D.; Zhang L. Revisiting the dispersion mechanism of grafted nanoparticles in polymer matrix: a detailed molecular dynamics simulation. Langmuir 2011, 27, 15213–15222. 10.1021/la203182u. 22040300

[ref41] Kulshreshtha A.; Jayaraman A. Dispersion and aggregation of polymer grafted particles in polymer nanocomposites driven by the hardness and size of the grafted layer tuned by attractive graft-matrix interactions. Macromolecules 2020, 53, 1302–1313. 10.1021/acs.macromol.9b02587.

[ref42] Goggin D. M.; Samaniuk J. R. 2D colloids: Size-and shape-controlled 2D materials at fluid–fluid interfaces. Langmuir 2021, 37, 14157–14166. 10.1021/acs.langmuir.1c02418. 34797659

[ref43] Konatham D.; Striolo A. Molecular design of stable graphene nanosheets dispersions. Nano Lett. 2008, 8, 4630–4641. 10.1021/nl802262p. 19367980

[ref44] Suter J. L.; Sinclair R. C.; Coveney P. V. Principles governing control of aggregation and dispersion of graphene and graphene oxide in polymer melts. Adv. Mater. 2020, 32, 2003213 10.1002/adma.202003213. 32720366

[ref45] Hsu D. D.; Xia W.; Arturo S. G.; Keten S. Systematic Method for Thermomechanically Consistent Coarse-Graining: A Universal Model for Methacrylate-Based Polymers. J. Chem. Theory Comput. 2014, 10, 2514–2527. 10.1021/ct500080h. 26580772

[ref46] Ruiz L.; Xia W.; Meng Z.; Keten S. A coarse-grained model for the mechanical behavior of multi-layer graphene. Carbon 2015, 82, 103–115. 10.1016/j.carbon.2014.10.040.

[ref47] Liao Y.; Molares Palmero O.; Arshad A.; Chen L.; Xia W. Molecular Dynamics Simulations of Crumpling Polymer-Grafted Graphene Sheets: Implications for Functional Nanocomposites. ACS Appl. Nano Mater. 2024, 7, 7802–7811. 10.1021/acsanm.4c00425.

[ref48] Chen X.; Zheng M.; Park C.; Ke C. Direct measurements of the mechanical strength of carbon nanotube–poly (methyl methacrylate) interfaces. Small 2013, 9, 3345–3351. 10.1002/smll.201202771. 23606544

[ref49] Yang Z.; Chiang C.-C.; Meng Z. Investigation of dynamic impact responses of layered polymer-graphene nanocomposite films using coarse-grained molecular dynamics simulations. Carbon 2023, 203, 202–210. 10.1016/j.carbon.2022.11.015. 36506702 PMC9731314

[ref50] Muhammad A.; Srivastava R.; Koutroumanis N.; Semitekolos D.; Chiavazzo E.; Pappas P.-N.; Galiotis C.; Asinari P.; Charitidis C. A.; Fasano M. Mesoscopic modeling and experimental validation of thermal and mechanical properties of polypropylene nanocomposites reinforced by graphene-based fillers. Macromolecules 2023, 56, 9969–9982. 10.1021/acs.macromol.3c01529. 38161324 PMC10753874

[ref51] Chiang C.-C.; Breslin J.; Weeks S.; Meng Z. Dynamic mechanical behaviors of nacre-inspired graphene-polymer nanocomposites depending on internal nanostructures. Extreme Mech. Lett. 2021, 49, 101451 10.1016/j.eml.2021.101451. 34541269 PMC8445040

[ref52] Wang Y.; Meng Z. Mechanical and viscoelastic properties of wrinkled graphene reinforced polymer nanocomposites–Effect of interlayer sliding within graphene sheets. Carbon 2021, 177, 128–137. 10.1016/j.carbon.2021.02.071. 33776064 PMC7990119

[ref53] Vallés C.; Papageorgiou D. G.; Lin F.; Li Z.; Spencer B. F.; Young R. J.; Kinloch I. A. PMMA-grafted graphene nanoplatelets to reinforce the mechanical and thermal properties of PMMA composites. Carbon 2020, 157, 750–760. 10.1016/j.carbon.2019.10.075.

[ref54] Wang Y.; Li Z.; Sun D.; Jiang N.; Niu K.; Giuntoli A.; Xia W. Understanding the thermomechanical behavior of graphene-reinforced conjugated polymer nanocomposites via coarse-grained modeling. Nanoscale 2023, 15, 17124–17137. 10.1039/D3NR03618A. 37850476

[ref55] Pal S.; Dansuk K.; Giuntoli A.; Sirk T. W.; Keten S. Predicting the Effect of Hardener Composition on the Mechanical and Fracture Properties of Epoxy Resins Using Molecular Modeling. Macromolecules 2023, 56, 4447–4456. 10.1021/acs.macromol.2c02577.

[ref56] Gurel U.; Keten S.; Giuntoli A. Bidispersity Improves the Toughness and Impact Resistance of Star-Polymer Thin Films. ACS Macro Lett. 2024, 13, 302–307. 10.1021/acsmacrolett.3c00671. 38373272 PMC10956491

[ref57] Alesadi A.; Xia W. Understanding the Role of Cohesive Interaction in Mechanical Behavior of a Glassy Polymer. Macromolecules 2020, 53, 2754–2763. 10.1021/acs.macromol.0c00067.

[ref58] Plimpton S. Fast Parallel Algorithms for Short-Range Molecular Dynamics. J. Comput. Phys. 1995, 117, 1–19. 10.1006/jcph.1995.1039.

[ref59] Michaud-Agrawal N.; Denning E. J.; Woolf T. B.; Beckstein O. MDAnalysis: a toolkit for the analysis of molecular dynamics simulations. J. Comput. Chem. 2011, 32, 2319–2327. 10.1002/jcc.21787. 21500218 PMC3144279

[ref60] Stukowski A. Visualization and Analysis of Atomistic Simulation Data with OVITO-the Open Visualization Tool. Modell. Simul. Mater. Sci. Eng. 2010, 18, 015012 10.1088/0965-0393/18/1/015012.

[ref61] Payne M. C.; Teter M. P.; Allan D. C.; Arias T. A.; Joannopoulos J. D. Iterative minimization techniques for ab initio total-energy calculations: molecular dynamics and conjugate gradients. Rev. Mod. Phys. 1992, 64, 1045–1097. 10.1103/RevModPhys.64.1045.

[ref62] Lü X.; Wu J.; Lin T.; Wan D.; Huang F.; Xie X.; Jiang M. Low-temperature rapid synthesis of high-quality pristine or boron-doped graphene via Wurtz-type reductive coupling reaction. J. Mater. Chem. 2011, 21, 10685–10689. 10.1039/c1jm11184a.

[ref63] Xia W.; Hsu D. D.; Keten S. Molecular Weight Effects on the Glass Transition and Confinement Behavior of Polymer Thin Films. Macromol. Rapid Commun. 2015, 36, 1422–1427. 10.1002/marc.201500194. 26033661

[ref64] Wan C.; Chen B. Reinforcement and interphase of polymer/graphene oxide nanocomposites. J. Mater. Chem. 2012, 22, 3637–3646. 10.1039/c2jm15062j.

[ref65] Krone M.; Stone J. E.; Ertl T.; Schulten K. Fast Visualization of Gaussian Density Surfaces for Molecular Dynamics and Particle System Trajectories. EuroVis - Short Papers 2012, 10, 067–071.

[ref66] Pramanik C.; Gissinger J. R.; Kumar S.; Heinz H. Carbon nanotube dispersion in solvents and polymer solutions: mechanisms, assembly, and preferences. ACS Nano 2017, 11, 12805–12816. 10.1021/acsnano.7b07684. 29179536

[ref67] McKechnie D.; Cree J.; Wadkin-Snaith D.; Johnston K. Glass transition temperature of a polymer thin film: Statistical and fitting uncertainties. Polymer 2020, 195, 122433 10.1016/j.polymer.2020.122433.

[ref68] Liao K.-H.; Aoyama S.; Abdala A. A.; Macosko C. Does graphene change Tg of nanocomposites?. Macromolecules 2014, 47, 8311–8319. 10.1021/ma501799z.

[ref69] Wang Y.; Li Z.; Niu K.; Xia W.; Giuntoli A. A. Molecular Dynamics Study of Mechanical and Conformational Properties of Conjugated Polymer Thin Films. Macromolecules 2024, 57, 5130–5142. 10.1021/acs.macromol.4c00232. 38882199 PMC11171455

[ref70] Gao Y.; Jing H. W.; Chen S. J.; Du M. R.; Chen W. Q.; Duan W. H. Influence of ultrasonication on the dispersion and enhancing effect of graphene oxide–carbon nanotube hybrid nanoreinforcement in cementitious composite. Compos. B: Eng. 2019, 164, 45–53. 10.1016/j.compositesb.2018.11.066.

[ref71] Peng S.; Yu Y.; Wu S.; Wang C.-H. Conductive polymer nanocomposites for stretchable electronics: material selection, design, and applications. ACS Appl. Mater. Interfaces 2021, 13, 43831–43854. 10.1021/acsami.1c15014. 34515471

[ref72] Payandehpeyman J.; Mazaheri M.; Khamehchi M. Prediction of electrical conductivity of polymer-graphene nanocomposites by developing an analytical model considering interphase, tunneling and geometry effects. Compos. Commun. 2020, 21, 100364 10.1016/j.coco.2020.100364.

[ref73] Zare Y.; Rhee K. Y. Development of a model for electrical conductivity of polymer/graphene nanocomposites assuming interphase and tunneling regions in conductive networks. Ind. Eng. Chem. Res. 2017, 56, 9107–9115. 10.1021/acs.iecr.7b01348.

[ref74] Klein D. J.; Randić M. Resistance distance. J. Math. Chem. 1993, 12, 81–95. 10.1007/bf01164627.

[ref75] Ellens W.; Spieksma F. M.; Van Mieghem P.; Jamakovic A.; Kooij R. E. Effective graph resistance. Linear Algebr. Appl. 2011, 435, 2491–2506. 10.1016/j.laa.2011.02.024.

[ref76] Spielman D. A.; Srivastava N. Graph sparsification by effective resistances Proceedings of the Fortieth Annual ACM Symposium on Theory of Computing; ACM, 2008, pp 563–568.

[ref77] Yang B.; Pan Y.; Yu Y.; Wu J.; Xia R.; Wang S.; Wang Y.; Su L.; Miao J.; Qian J.; et al. Filler network structure in graphene nanoplatelet (GNP)-filled polymethyl methacrylate (PMMA) composites: From thermorheology to electrically and thermally conductive properties. Polym. Test. 2020, 89, 106575 10.1016/j.polymertesting.2020.106575.

[ref78] Pan Y.; Li L.; Chan S. H.; Zhao J. Correlation between dispersion state and electrical conductivity of MWCNTs/PP composites prepared by melt blending. Compos. A: Appl. Sci. Manuf. 2010, 41, 419–426. 10.1016/j.compositesa.2009.11.009.

[ref79] Gao K.; Huang Y.; Han Y.; Gao Y.; Dong C.; Liu J.; Li F.; Zhang L. Designing Heterogeneous Surfaces of Two-Dimensional Nanosheets to Maximize Mechanical Reinforcing of Polymer Nanocomposites via Molecular Dynamics Simulation. Macromolecules 2022, 55, 6620–6632. 10.1021/acs.macromol.2c00375.

