# Supplementary material for: Optimizing Graphene Dispersion via Polymer Grafting
Source: Macromolecules. 2025 Jan 2;58(5):2224–36. doi: 10.1021/acs.macromol.4c02249 (PMC11912542; doi:10.1021/acs.macromol.4c02249)
Supplement: Supplementary file 1 — ma4c02249_si_001.pdf [file ma4c02249_si_001.pdf]

Supporting Information for Optimizing Graphene Dispersion via Polymer Grafting

Yang Wang,[†,‡] Wenjie Xia,[*,¶] and Andrea Giuntoli[*,†]

†*Zernike Institute for Advanced Materials, University of Groningen, 9747 AG, Groningen, The Netherlands*

‡*Department of Theoretical Physics & Center for Biophysics, Saarland University, 66123 Saarbrücken, Germany*

¶*Department of Aerospace Engineering, Iowa State University, Ames, IA 50011, United States*

E-mail: wxia@iastate.edu; a.giuntoli@rug.nl

Details of the CG models

We implement the atomistically informed coarse-grained (CG) models of graphene and poly(methyl methacrylate) p(MMA) to simulate graphene/p(MMA) nanocomposites for improving computational efficiency. The CG models of graphene and p(MMA) are developed by Xia et al.[1,2] using the strain energy conservation and iterative Boltzmann inversion approaches, respectively. The coarse-grained mapping schemes of the p(MMA) and graphene CG models are depicted in **Figure 1** in the main text. The masses of the CG bead types A, B, and C are 48 g/mol, 85.1 g/mol, and 15 g/mol, respectively. The CG models for p(MMA) and graphene have improved computational speed and access to larger spatiotemporal scales compared to all-atomistic (AA) simulations. These models have been utilized in studies of multilayer graphene assemblies,[3] graphene melt,[4] graphene-reinforced polymers,[5–8] crumpled systems,[9] etc. The relative CG bonded and nonbonded potentials and parameters are summarized in **Table S1** and **S2**. We use the Lorenz–Berthelot mixing rule for describing the nonbonded interactions between graphene and p(MMA), which is commonly used in the polymer nanocomposites:[10,11]

$$\sigma_{ij} = \frac{\sigma_{ii} + \sigma_{jj}}{2} \tag{1}$$

$$\epsilon_{ij} = \sqrt{\epsilon_{ii} \cdot \epsilon_{jj}} \tag{2}$$

where i and j denote different CG bead types.

Simulation Protocol

It is noted that the final configuration and dispersion state of graphene are highly dependent on the initial configuration and simulation protocol. In our previous work on graphene-reinforced poly(3-alkylthiophene) (P3AT) nanocomposites,[12] due to obstruction by the surrounding polymers, graphene exhibits limited displacement in dense systems based on the analysis of the root-mean-square deviation (RMSD) of the center of mass of graphene. Although it is currently impossible to simulate timescales comparable to experiments, one can speculate that graphene sheets would be susceptible to aggregate over an infinitely long simulation. This occurs because the interactions between graphene sheets are stronger than those between graphene and the polymer and the planar geometry of graphene. Once "naked" graphene sheets encounter each other in the system, they will bind together. In addition, pristine graphene indeed tends to aggregate easily in experiments.[13] Thus, to accelerate the simulation and represent the aggregation behavior of pristine graphene as observed in experiments, we implement a purely repulsive pair interaction between p(MMA) chains.

As shown in **Figure S1**, the LJ potential of the nonbonded pairs B-B, C-C, and B-C is truncated at $2^{1/6} * \sigma_{BB}$, $2^{1/6} * \sigma_{CC}$, and $2^{1/6} * \sigma_{BC}$ and then shifted to zero. In addition, the full LJ potential is utilized for the nonbonded pairs of A-B, A-C, and A-A. In this way, the p(MMA) can be absorbed on the graphene surface and graphene sheets can be aggregated during the simulation, hindered only by the steric interactions of the grafted chains.

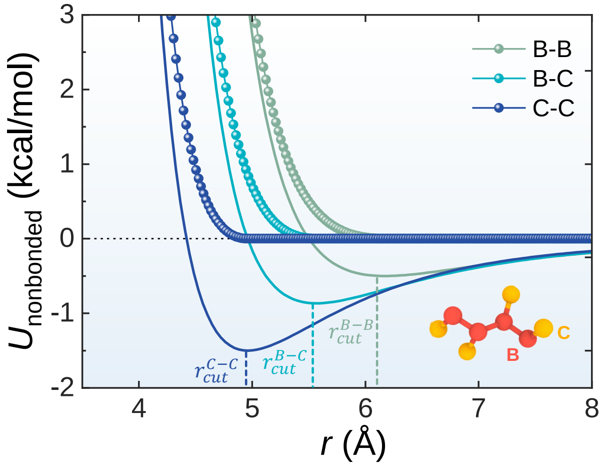

Figure S1. The full (solid line) and repulsive (line and symbol) LJ potential curves of the CG bead B and C of p(MMA). The repulsive LJ potential is obtained by shifting the full LJ curve to zero at its cutoff distance $2^{\frac{1}{6}} * \sigma$, corresponding to the position where $U_{non-bonded}$ equals zero (vertical dashed line).

To model nanocomposites, we begin by constructing p(MMA)-grafted graphene building blocks (**Figure 2** in the main text). Subsequently, 50 of these building blocks are randomly distributed in a large box using an in-house Python script, and some isolated p(MMA) chains with 80 monomers per chain are randomly inserted into the simulation box to ensure the total number of CG beads in the system (**Table 1** in the main text). To start the simulation, an initial velocity based on a temperature of 800 K is used only for all CG beads to initialize the structure followed by the energy minimization using the iterative conjugant gradient algorithm.[14] The timestep used in the simulation is 4 fs, with periodic boundary conditions (PBC) applied in all three directions. To improve the simulation, the system is deformed to reach a moderate density, *i.e.*, 0.3 g/cm^3, as the initial configuration. Subsequently, guided by the repulsive interactions described above, the nanocomposite system undergoes relaxation under the NVT ensemble (constant volume, constant temperature) at 800 K for a duration of 84 ns. Then, we turn on the NPT ensemble, and the system is gradually cooled down to 300 K and condensed over 4 ns followed by another 4 ns equilibration to stabilize the system at 300 K. Lastly, equilibration runs lasting 0.4 ns are conducted to sample the trajectory. **Figure S2** illustrates the dispersion state of graphene over simulation time. It is

noted that the dispersion parameter $f_d(t)$ exhibits a sharp increase at $t = 84$ ns, coinciding with the transition from the NVT to the NPT ensemble, during which graphene sheets approach each other as the system condenses under the NPT ensemble.

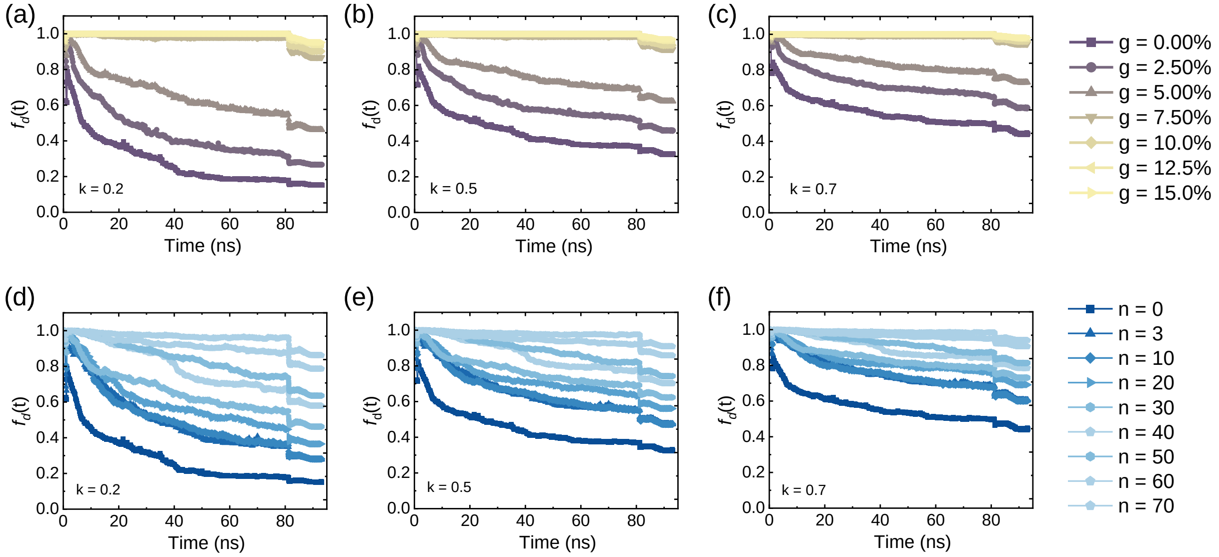

Figure S2. Dispersion parameter $f_d(t)$ as a function of simulation time with different coefficients k as shown in **Eq. 3** in the main text.

Dispersion analysis

Figure S2 shows the $f_d(t)$ curves under varying k values as a function of simulation time for the graphene/p(MMA) nanocomposites with different grafting densities and grafted chain lengths. The dispersion state of graphene, *i.e.*, $[f_A, f_I, f_U]$, is sampled from the last 0.4 ns equilibration stage and plotted in **Figures 3**c and f in the main text. The results demonstrate a significant change in $f_d(t)$ with increasing grafting density and grafted chain length of p(MMA) onto the graphene surface at the final MD stage (**Figures 3**b and e in the main text). The detailed dispersion state, $[f_A,\ f_I,\ f_U]$, of each graphene sheet within the nanocomposite is summarized in **Tables S3** and **S4**, where each $[f_A,\ f_I,\ f_U]$ denotes one data point in **Figures 3**c and f. It is important to note that if two graphene sheets are aggregated, their dispersion state is identical, *i.e.*, $[f_A,\ f_I,\ f_U] = [1,\ 0,\ 0]$, and the number of such aggregated graphene sheets is even, as indicated by the data in bold font in **Table S3**. Additionally, the dispersion state f_A is nearly zero for graphene/p(MMA) nanocomposites with $g > 10\%$.

To better understand the effect of the purely repulsive interaction between p(MMA) on the final graphene configuration within the polymer matrix, we conducted a CGMD sim-

ulation using the same protocol but with the entire LJ potential (the solid line in **Figure S1**) between p(MMA) for the graphene/p(MMA) nanocomposite with $g = 0.00\%$. All other simulation steps remained unchanged. **Figures S3**a and b present snapshots of the final graphene morphology in graphene/p(MMA) nanocomposite systems, comparing one system that incorporates repulsive interactions between p(MMA) during dynamics (**Figure S3**a) and another that does not (**Figure S3**b). The corresponding dispersity parameter $f_d(t)$ as a function of simulation time is depicted in **Figure S3**c, and the final averaged $\bar{f}_d$ is shown in **Figure S3**d. Results demonstrate that the interactions between polymers significantly influence the final morphology of graphene. In the nanocomposite using the LJ potential without repulsive interactions between p(MMA), polymer chains tend to adhere to nearby graphene, forming a shell around the graphene sheet in the initial stages of simulation, thereby inhibiting graphene-to-graphene contact as the simulation progresses. A key choice we made is to use a short cutoff potential for the polymer-polymer non-bonded interactions, leading to pure repulsive behavior and faster dynamics of p(MMA) chains. Polymer-graphene and graphene-graphene interactions remain attractive, with the competing energy scales favoring graphene aggregation when graphene sheets are pristine instead of grafted with chains. This aggregation of pristine graphene is in line with the expected equilibrium behavior of the composite both from the interactions of the model and from experimental observations,[15,16] but attractive polymer-graphene interactions would like to chains bound to the graphene sheets that would then take a very long time to diffuse and equilibrate, past the timescales accessible to our simulations. By making this choice, we do not affect the equilibrium state of the model and we accelerate the system equilibration. As shown in **Figure S3**b, if we choose the attractive polymer-graphene LJ potential, the graphene sheets in the system show a good dispersion state, i.e., $\bar{f}_d = 0.65$, (the blue graphene CG beads, unbound morphology in the system) simply because the graphene sheets do not have time to diffuse and aggregate, as also evidenced by the root mean-square-displacement of graphene in one of our recent papers on graphene composites where out-of-equilibrium, glassy states were investigated.[17] In a recent study on graphene/polypropylene nanocomposites, the author employed the entire LJ potential, resulting in no significant graphene aggregation.[18] Hence, the purely repulsive interaction within the polymer emerges as the pivotal factor driving the aggregated morphology of graphene clusters in pristine graphene/polymer nanocomposites, which should be carefully considered when simulating nanofiller-reinforced composites.

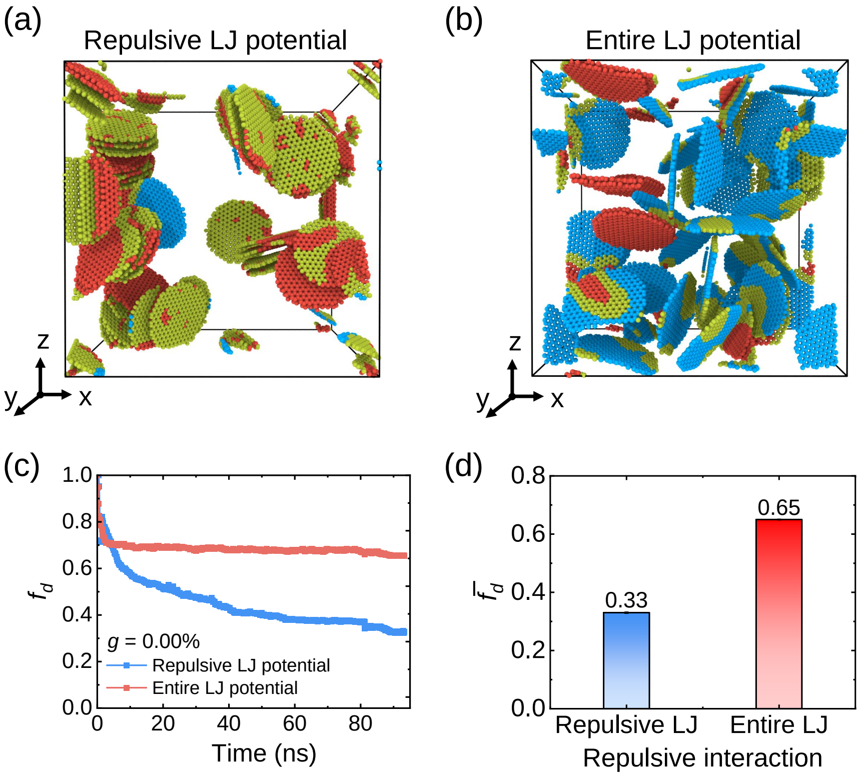

Figure S3. The snapshots of the final graphene morphology of the graphene/p(MMA) nanocomposite system ($g = 0.00\%$) (a) with and (b) without purely repulsive interactions between p(MMA) during dynamics. The p(MMA) chains are omitted for clarity. Graphene CG beads colored in red, green, and blue represent the aggregated, intercalated, and unbound states, respectively, as illustrated in **Figure 3** in the main text. (c) The $f_d(t)$ vs. simulation time curves for the graphene/p(MMA) nanocomposites are compared with and without repulsive interactions, while (d) shows the equilibrated $\bar{f}_d$ for both scenarios.

Figure S4 demonstrates the Gaussian density surface distribution on graphene clusters and the definition of aggregation energy, *i.e.*, $E_{Aggregation}$. The Gaussian density surface is calculated by the OVITO Python package,[19] which calculates an isosurface from a volumetric Gaussian density map derived from the atoms or particles in the vicinity of each lattice point,[20] as shown in **Figure S4**a. The Gaussian density surface of the graphene/p(MMA) nanocomposite with $g = 0.00\%$ is depicted in **Figure S4**b. To characterize the effect of the dispersion state of graphene on the aggregation energy, we constructed two laminated graphene/p(MMA) nanocomposites with three graphene sheets: one with perfectly dispersed graphene sheets (**Figure S4**c) and another with perfectly stacked graphene sheets (**Figure S4**d). As shown in **Figure S4**c, the distance between adjacent graphene sheets in the dispersed configuration is 6 nm, significantly greater than the cutoff distance, indicating no interaction energy between the graphene sheets. The $E_{Aggregation}$ values for the two cases are derived as 0.335 and 0.292 J/m^2, respectively, revealing the lower $E_{Aggregation}$ value for the

nanocomposite system with good graphene dispersity. Additionally, we performed the same simulation with four graphene sheets embedded in the laminated nanocomposite, the yielding $E_{Aggregation}$ values are 0.293 and 0.334 J/m^2 for the dispersed and stacked configurations, respectively.

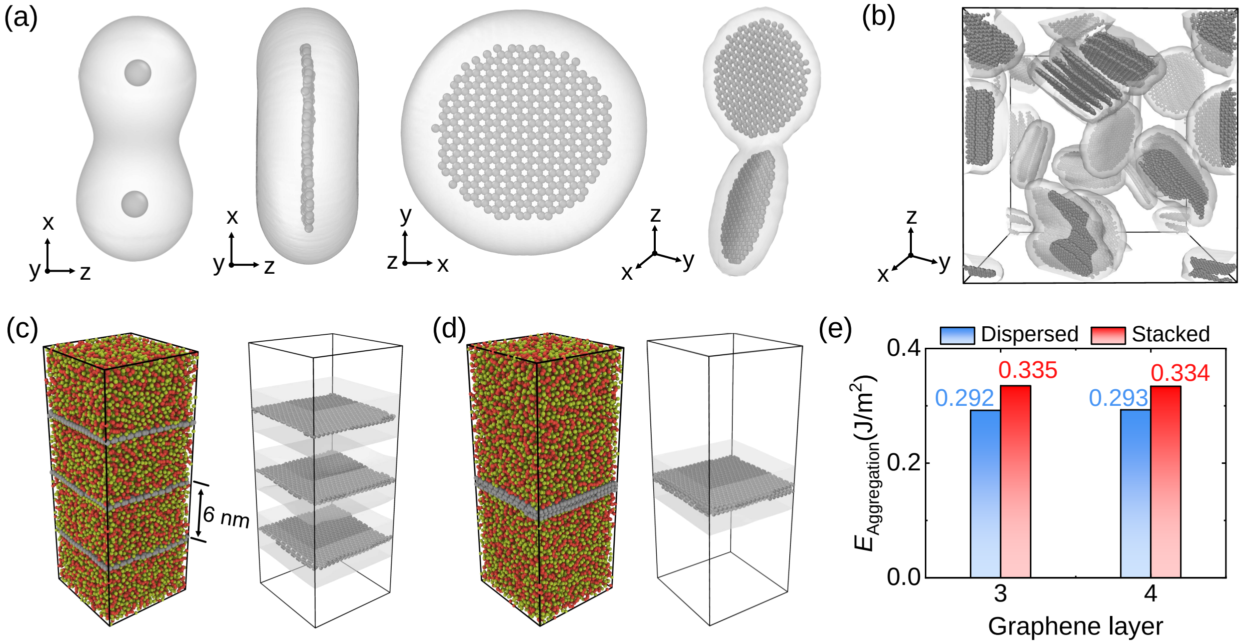

Figure S4. Illustration of the Gaussian density surface of (a) CG bead, graphene sheet, and (b) graphene clusters in the graphene/p(MMA) nanocomposites with $g = 0.00\%$, where the p(MMA) chains are omitted for clarity. The equilibrated system of graphene/p(MMA) laminated nanocomposites with (c) perfectly dispersed graphene and (d) stacked graphene. The right panels of (c) and (d) represent the Gaussian density surface of the graphene sheet. (e) The averaged $E_{Aggregation}$ for the dispersed and stacked graphene/p(MMA) laminated nanocomposites with varying numbers of embedded graphene layers.

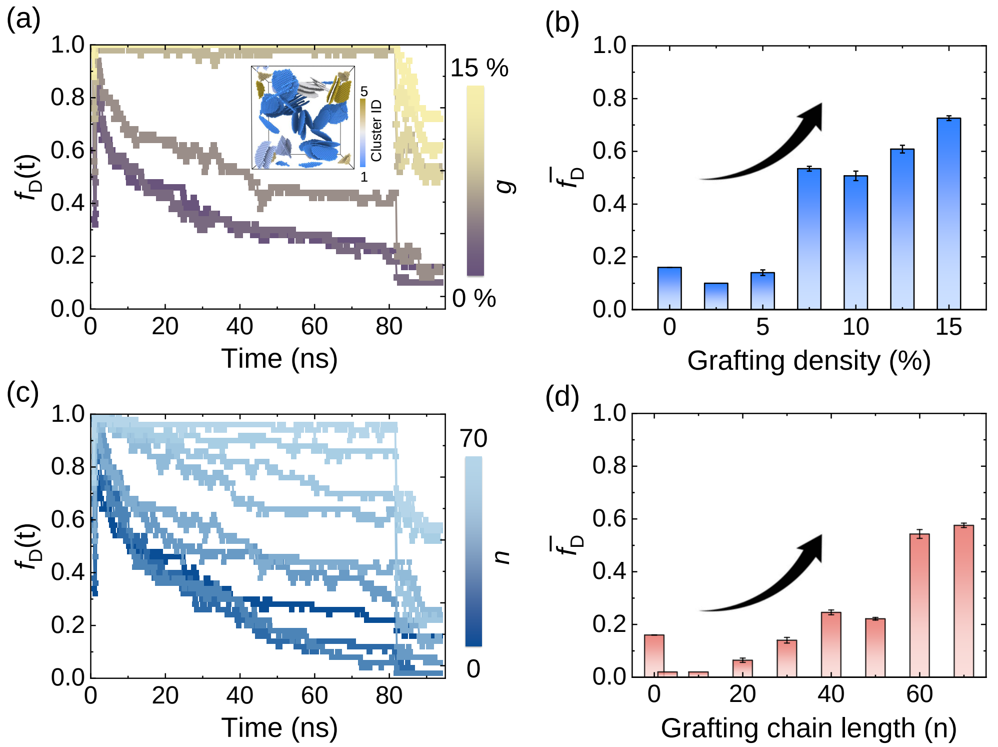

Figure S5. The degree of dispersion f_D as a function of simulation time for the graphene/p(MMA) nanocomposites with (a) different grafting densities and (c) grafted chain lengths. The averaged $\bar{f}_D$ in the last 0.4 ns simulation stage for the graphene/p(MMA) nanocomposites with (b) different grafting densities and (d) grafted chain lengths.

In addition, we introduce another method to characterize the graphene morphology based on the distribution of nearest neighbor distances for CG graphene flake atoms. Cha et al.[21] proposed that when the minimum separation between CG beads on two different graphene flakes is less than 3.5*σ_{AA}, the flakes are considered agglomerated and treated as a single cluster. They also introduced a parameter, f_D, to quantify the degree of dispersion, ranging from 0 to 1:

$$f_D = \frac{G_{act}}{N_{Total}} \tag{3}$$

$$G_{act} = N_{Total} - \sum_{i,j}(m_i - 1) \cdot n_j \tag{4}$$

where N_{Total} is the total number of graphene sheets in the composite, G_{act} is the number of active graphene sheets, which is determined by subtracting the number of graphene sheets that have agglomerated to form clusters from the total number of graphene sheets. Specifically, n_j is the number of clusters composed of m_i aggregated graphene sheets. It is noted that the large f_D value indicates good graphene dispersity, which is different from the quantity of f_d in the main text. The inset in **Figure S5**a shows the graphene cluster

with $g = 0.0\%$. **Figures S5**a and c show the evolution of f_D as a function of simulation time for the graphene/p(MMA) systems with different grafting densities and grafted chain lengths, and the equilibrated f_D values are shown in **Figures S5**b and d. Generally, it is observed that the averaged $\bar{f_D}$ in the equilibrated system increases with higher grafting density and longer grafted chain length of p(MMA) on the graphene surface (**Figures S5**b and d), indicating good dispersity of graphene. However, there are fluctuations in $\bar{f_D}$ with respect to grafting density and grafted chain length. These fluctuations arise because $\bar{f_D}$ considers the distance between different graphene sheets, treating edge-to-edge contacts of graphene sheets as aggregation, which is a rough approximation compared to the definition of f_d as illustrated in the main text.

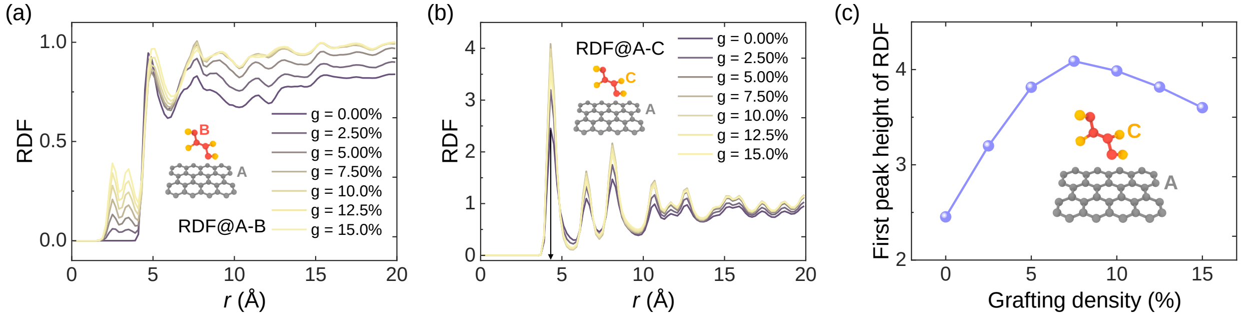

Figure S6. Radial distribution functions (RDFs) between (a) CG beads A and B, and (b) between CG beads A and C of graphene sheets in the graphene/p(MMA) nanocomposites with different grafting densities. (c) The first peak height of the RDF curves between CG beads A and C.

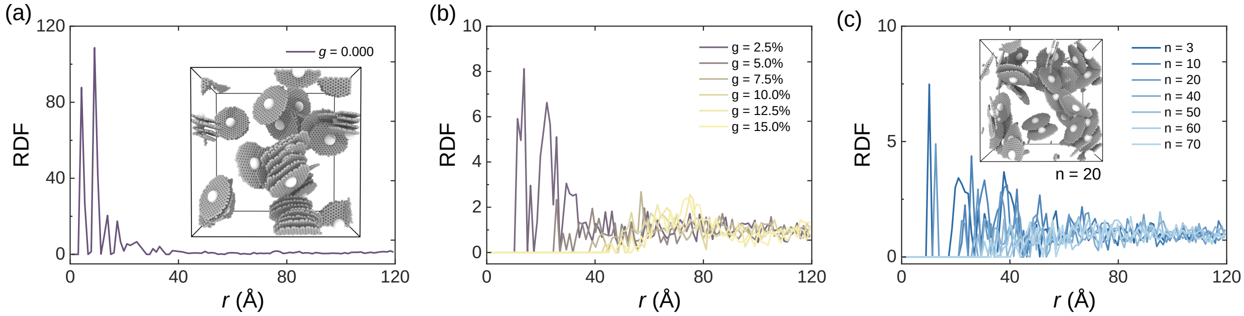

Figure S7. Radial distribution functions (RDFs) of the center-of-mass of graphene sheets in the graphene/p(MMA) nanocomposites with different (a, b) grafting densities and (c) grafted chain lengths. The insets in panels (a) and (c) show the snapshot of graphene/p(MMA) nanocomposite with $g = 0.00\%$ and $n = 20$. The white beads represent the center-of-mass of the graphene, and the p(MMA) chains are omitted for clarity.

Structure property

In **Figure 4**c in the main text, the $E_{Interfacial}$ of graphene/p(MMA) nanocomposites with different grafting densities represents a slight decrease as $g > 7.5\%$, which we attribute to the insufficient contact between graphene and p(MMA). **Figures S6**a and b show the radial distribution functions (RDFs) of the pairs of A-B and A-C under varying grafting densities. Results show that for $g = 0.00\%$, the RDF curve between beads A and B represents the first peak at the location of 4.7 Å (**Figure S6**a). Upon grafting p(MMA) chains onto the graphene sheet, the RDF between beads A and B reveals two distinct peaks at 2.5 Å and 3.5 Å. This phenomenon arises due to the covalent bonding between bead A and bead B. In addition, the RDF curves between CG beads A and C are similar under varying grafting densities but slightly different amplitudes of the peaks. **Figure S6**c displays the first peak height of the RDF between CG beads A and C as a function of grafting densities. The peak value decreases when $g > 0.00\%$, due to the obstruction caused by high grafting density, which leads to insufficient contact between p(MMA) and graphene. Additionally, **Figure S7** presents the RDF between the center-of-mass of graphene sheets in graphene/p(MMA) nanocomposites with varying grafting densities and grafted chain lengths. The results indicate that the RDF peak is more pronounced for the graphene/p(MMA) with $g = 0.00\%$ compared to the graphene/p(MMA) with $n = 20$, indicating highly aggregated graphene and poor dispersity. As the grafting density and grafted chain length increase, the sharp peaks of the RDF curve within the range of 0-20 Å disappear, indicating improved dispersity of the graphene.

Thermal, Dynamic, and Mechanical Properties

Figure S8a and b display the glass transition temperatures (T_g) of graphene/p(MMA) nanocomposite systems: $g = 5.00\%$ and $n = 70$, $g = 5.00\%$ and $n = 30$, and $g = 15.00\%$ and $n = 30$. The results indicate an enhancement in T_g compared to the pristine p(MMA) system (385.2 ± 1.9 K),[1] with no significant change in T_g for varying g and n. **Figure S9** illustrates the mean-square-displacement (MSD) of graphene/p(MMA) nanocomposites with different grafting densities and chain lengths, showing a slight decrease in MSD with increasing grafting density and chain length. Previous studies have established that the Debye-Waller factor (DWF) $\langle u^2 \rangle$ measures segmental "rattle-space" at the picosecond scale, providing information on local mobility and free volume. Accordingly, local molecular stiffness is described as $1/\langle u^2 \rangle$.[12] Based on previous work on p(MMA) thin films using the same coarse-grained (CG) model, $\langle u^2 \rangle$ is obtained from MSD measurements at $t = 10$ ps, corresponding to the dislocation time marking the transition from ballistic to caged motion.

To better illustrate molecular stiffness variations within the nanocomposite system, three-dimensional (3D) color maps of local molecular stiffness $1/\langle u^2\rangle$ of the CG beads are shown in **Figure S9**c and S10d, using a cubic cell size of 5 Å for the calculations. The results reveal significant dynamic heterogeneity within the system, with graphene sheets displaying the highest $1/\langle u^2\rangle$, as indicated by the red color in the 3D plot. Additionally, **Figures S9**e and f present the $1/\langle u^2\rangle$ of graphene sheets in graphene/p(MMA) nanocomposites with $g = 0.00\%$ and $n = 20$, respectively. The results indicate that molecular stiffness is not homogeneous on the graphene sheet, likely due to the fluctuating properties of the sheets and different grafting scenarios on each graphene surface.

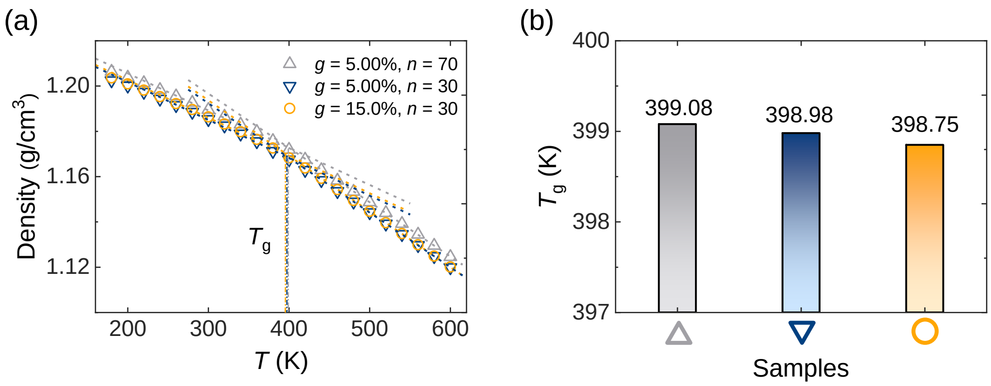

Figure S8. (a) Density versus temperature curves for graphene/p(MMA) nanocomposites with varying grafted chain lengths and grafting densities. The dashed lines represent the linear fits for density in the low (200 - 300 K) and high (500 - 600 K) temperature ranges, and the vertical dashed lines represent the glass transition temperature (T_g). (b) T_g values of the three systems depicted in panel (a).

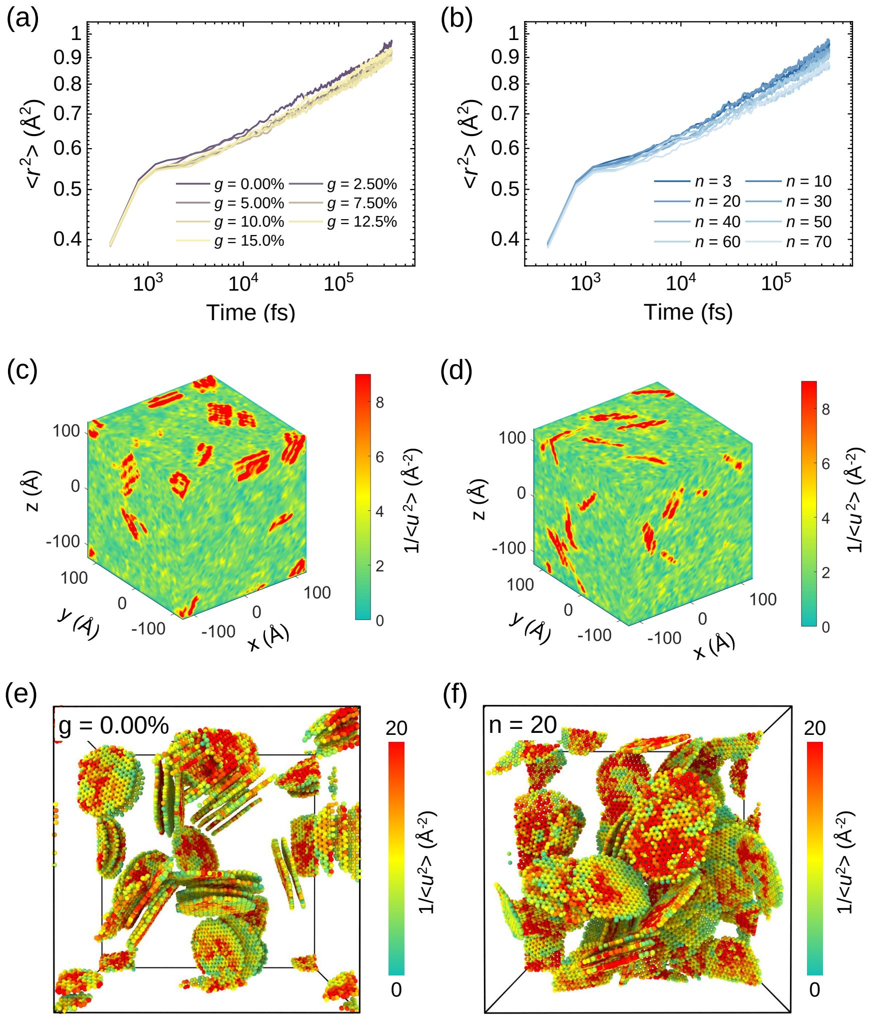

Figure S9. Mean-square-displacement of graphene/p(MMA) nanocomposites with different (a) grafting densities and (b) grafted chain lengths. (c) Color maps of local molecular stiffness $1/\langle u^2\rangle$ of all CG beads in the graphene/p(MMA) nanocomposite systems with (c) $g = 0.0\%$ and (d) $n = 20$. Typical visualization of local molecular stiffnesses $1/\langle u^2\rangle$ for the CG beads of graphene sheets in the graphene/p(MMA) nanocomposite systems with (e) $g = 0.0\%$ and (f) $n = 20$.

Percolation and Conductivity

In this study, we set the tunneling distance (electrical threshold) for the graphene sheet as 2 nm.[22] We then retain the graphene sheets that percolate in all three directions and eliminate the small, isolated graphene clusters. As shown in **Figure S10**, we calculate the center of mass of the graphene sheets in the percolated network, designate these points as nodes, and connect the nodes with edges to form a graph network, which is further used to

calculate electrical conductivity as elucidated in the main text. Different graphene sheets are depicted in various colors. It's important to note that an edge will only form between two graphene sheets when the distance between CG beads in the two sheets is less than 2 nm. However, in the two-dimensional plot, the edges are constrained within the box and not through the boundary, causing some edges to appear disproportionately long.

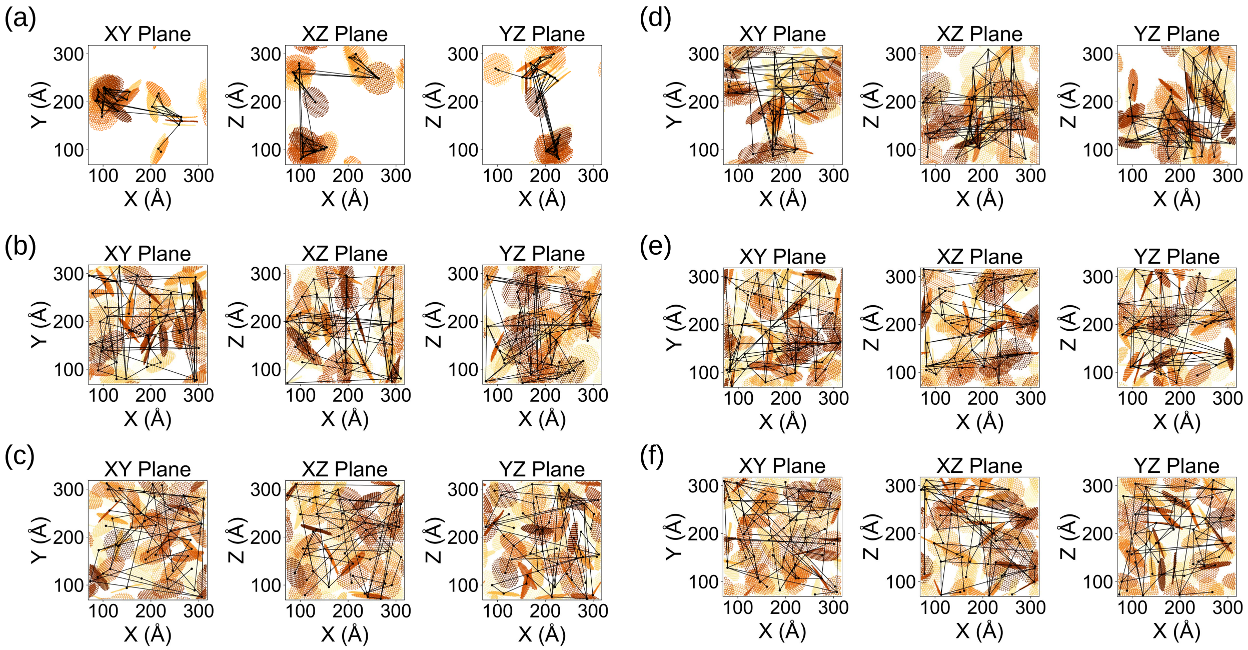

Figure S10. The percolation network of graphene sheets within the graphene/p(MMA) nanocomposites with different grafting densities, *i.e.*, (a) g = 0.00%, (b) g = 5.00%, and (c) g = 12.5%, and with different grafted chain lengths, *i.e.*, (d) n = 20, (e) n = 50, (f) n = 70. The graphene sheets are colored in different colors. The black dots represent the centers of mass of the graphene sheets, and the black solid lines connect the center-of-masses of graphene sheets that meet the threshold distance of 2 nm, meaning the edge is formed when the minimum distance between beads in two graphene sheets is less than 2 nm.

Table S1. Functional Forms and Parameters of Bond, Angle, and Dihedral Interactions for CG Model of p(MMA) and graphene models.[1,2] 'A', 'B', and 'C' denote the graphene CG beads, p(MMA) backbone beads, and p(MMA) sidechain beads, respectively.

Interaction	Potential form	Parameters
B-B bond	$U_{\text{bond}}(l) = k_b(l - l_0)^2$	$k_b = 105.0$ kcal/mol $\cdot$ Å^2, $l_0 = 2.735$ Å.
B-C bond		$k_b = 39.86$ kcal/mol $\cdot$ Å^2, $l_0 = 3.658$ Å.
A-A bond	$U_{bond}(l) = D_0[1 - e^{-\alpha(d-d_0)}]^2$, for $d < d_{cut}$.	$d_0 = 2.8$ Å, $D_0 = 196.38$ kcal/mol $\alpha = 1.55$ Å^{-1}, $d_{cut} = 3.25$ Å.
B-B-B angle	$U_{\text{angle}}(\theta) = -k_B T \ln \left\{ \sum_{i=1}^{2} \left[a_i \cdot \exp\left(\frac{-(\theta-\theta_0)^2}{b_i} \right) \right] \right\}$	$a_1 = 2.294 \times 10^{-2}$, $b_1 = 9.493°$, $\theta_1 = 121.0°$, $a_2 = 4.367 \times 10^{-3}$, $b_2 = 6.210°$, $\theta_2 = 158.5°$.
B-B-C angle	$U_{\text{angle}}(\theta) = \sum_{i=2}^{4} k_i \cdot (\theta - \theta_0)^i$	$k_2 = 9.881$ kcal/mol $\cdot$ rad^2, $k_3 = -15.12$ kcal/mol $\cdot$ rad^3, $k_4 = 6.589$ kcal/mol $\cdot$ rad^4.
A-A-A angle	$U_{\text{angle}}(\theta) = k_\theta \cdot (\theta - \theta_0)^2$	$\theta_0 = 120°, k_\theta = 409.4$ kcal/mol.
B-B-B-B dihedral	$U_{\text{dihedral}}(\phi) = \sum_{i=1}^{5} \left[a_i \cdot \cos^{i-1}(\phi) \right]$	$a_1 = 4.380$ kcal/mol, $a_2 = 8.739 \times 10^{-1}$ kcal/mol, $a_3 = -3.571 \times 10^{-1}$ kcal/mol, $a_4 = -2.774 \times 10^{-1}$ kcal/mol, $a_5 = 9.321 \times 10^{-2}$ kcal/mol.
C-B-B-C dihedral	$U_{\text{dihedral}}(\phi) = \sum_{i=1}^{5} \left[a_i \cdot \cos^{i-1}(\phi) \right]$	$a_1 = 4.519$ kcal/mol, $a_2 = -8.859 \times 10^{-1}$ kcal/mol, $a_3 = -1.692$ kcal/mol, $a_4 = -5.625 \times 10^{-1}$ kcal/mol, $a_5 = 9.562 \times 10^{-2}$ kcal/mol.
A-A-A-A dihedral	$U_{\text{dihedral}}(\phi) = k_\phi \left[1 - \cos(2\phi)\right]$	$k_\phi = 4.15$ kcal/mol

Table S2. Nonbonded interactions for p(MMA) and graphene CG models.[1,2] 'A', 'B', and 'C' denote the graphene CG beads, p(MMA) backbone beads, and p(MMA) sidechain beads, respectively.

Potential form	Parameters
$E_{LJ}(r) = 4\epsilon \left[(\frac{\sigma}{r})^{12} - (\frac{\sigma}{r})^6 \right]$	$\epsilon_{AA} = 0.821$ kcal/mol, $\sigma_{AA} = 3.46$ Å $\epsilon_{BB} = 0.500$ kcal/mol, $\sigma_{BB} = 5.50$ Å $\epsilon_{CC} = 1.500$ kcal/mol, $\sigma_{CC} = 4.42$ Å

Table S3. The graphene dispersion state of $[f_A,\ f_I,\ f_U]$ for graphene/p(MMA) nanocomposites with grafting densities of g = 5%, 10%, and 15%, respectively. f_A, f_I, and f_U represent the percentage of graphene beads in the dispersion state of Aggregated, Intercalated, and Unbound for each graphene. The data in bold font indicates that the entire graphene sheet is in an Aggregated state, as the red color graphene shown in main text **Figure 3**a.

g	$g = 0.0\%$	$g = 5.0\%$	$g = 10\%$	$g = 15\%$
Graphene ID	$[f_A,\ f_I,\ f_U]$	$[f_A,\ f_I,\ f_U]$	$[f_A,\ f_I,\ f_U]$	$[f_A,\ f_I,\ f_U]$
1	[0.089, 0.911, 0.000]	[0.043, 0.616, 0.341]	[0.000, 0.033, 0.967]	[0.000, 0.101, 0.899]
2	[**1.000, 0.000, 0.000**]	[0.087, 0.838, 0.075]	[0.006, 0.213, 0.781]	[0.000, 0.030, 0.970]
3	[0.745, 0.255, 0.000]	[0.051, 0.452, 0.497]	[0.000, 0.170, 0.830]	[0.000, 0.002, 0.998]
4	[0.311, 0.689, 0.000]	[0.444, 0.211, 0.345]	[0.000, 0.012, 0.988]	[0.000, 0.022, 0.978]
5	[0.230, 0.770, 0.000]	[0.075, 0.905, 0.020]	[0.000, 0.045, 0.955]	[0.000, 0.039, 0.961]
6	[0.083, 0.917, 0.000]	[0.000, 0.172, 0.828]	[0.000, 0.222, 0.778]	[0.000, 0.055, 0.945]
7	[0.895, 0.105, 0.000]	[0.042, 0.747, 0.211]	[0.005, 0.071, 0.924]	[0.000, 0.000, 1.000]
8	[0.328, 0.672, 0.000]	[0.063, 0.752, 0.186]	[0.000, 0.081, 0.919]	[0.000, 0.057, 0.943]
9	[0.161, 0.804, 0.035]	[0.003, 0.100, 0.897]	[0.007, 0.295, 0.699]	[0.000, 0.076, 0.924]
10	[0.194, 0.806, 0.000]	[0.077, 0.697, 0.226]	[0.000, 0.130, 0.870]	[0.000, 0.039, 0.961]
11	[**1.000, 0.000, 0.000**]	[0.178, 0.565, 0.256]	[0.000, 0.027, 0.973]	[0.000, 0.015, 0.985]
12	[0.117, 0.883, 0.000]	[0.052, 0.770, 0.179]	[0.000, 0.073, 0.927]	[0.000, 0.059, 0.941]
13	[0.032, 0.646, 0.322]	[0.079, 0.565, 0.356]	[0.000, 0.032, 0.968]	[0.000, 0.000, 1.000]
14	[0.114, 0.886, 0.000]	[0.000, 0.000, 1.000]	[0.000, 0.035, 0.965]	[0.000, 0.045, 0.955]
15	[**1.000, 0.000, 0.000**]	[0.205, 0.233, 0.562]	[0.000, 0.153, 0.847]	[0.000, 0.032, 0.968]
16	[**1.000, 0.000, 0.000**]	[0.004, 0.244, 0.752]	[0.000, 0.123, 0.877]	[0.000, 0.017, 0.983]
17	[0.211, 0.789, 0.000]	[0.125, 0.724, 0.151]	[0.000, 0.000, 1.000]	[0.000, 0.050, 0.950]
18	[0.563, 0.428, 0.009]	[0.038, 0.571, 0.391]	[0.000, 0.043, 0.957]	[0.000, 0.005, 0.995]
19	[0.216, 0.429, 0.355]	[0.061, 0.683, 0.257]	[0.007, 0.167, 0.825]	[0.000, 0.091, 0.909]
20	[0.472, 0.528, 0.000]	[0.027, 0.592, 0.381]	[0.000, 0.000, 1.000]	[0.000, 0.002, 0.998]
21	[0.586, 0.414, 0.000]	[0.288, 0.195, 0.518]	[0.014, 0.297, 0.689]	[0.000, 0.016, 0.984]
22	[0.066, 0.934, 0.000]	[0.033, 0.952, 0.015]	[0.000, 0.126, 0.874]	[0.000, 0.032, 0.968]
23	[0.156, 0.844, 0.000]	[0.243, 0.727, 0.029]	[0.000, 0.065, 0.935]	[0.000, 0.049, 0.951]
24	[**1.000, 0.000, 0.000**]	[0.276, 0.716, 0.007]	[0.000, 0.010, 0.990]	[0.000, 0.013, 0.987]
25	[0.765, 0.235, 0.000]	[0.136, 0.587, 0.276]	[0.019, 0.155, 0.826]	[0.000, 0.013, 0.987]
26	[0.100, 0.848, 0.052]	[0.188, 0.546, 0.266]	[0.014, 0.234, 0.752]	[0.000, 0.045, 0.955]
27	[0.102, 0.898, 0.000]	[0.027, 0.391, 0.582]	[0.005, 0.132, 0.864]	[0.000, 0.169, 0.831]
28	[0.557, 0.443, 0.000]	[0.093, 0.816, 0.091]	[0.000, 0.151, 0.849]	[0.000, 0.128, 0.872]
29	[0.099, 0.901, 0.000]	[0.214, 0.265, 0.521]	[0.000, 0.096, 0.904]	[0.000, 0.052, 0.948]
30	[0.178, 0.822, 0.000]	[0.138, 0.586, 0.276]	[0.000, 0.090, 0.910]	[0.000, 0.218, 0.782]
31	[0.069, 0.931, 0.000]	[0.086, 0.556, 0.358]	[0.000, 0.090, 0.910]	[0.000, 0.084, 0.916]
32	[0.141, 0.859, 0.000]	[0.478, 0.254, 0.269]	[0.000, 0.093, 0.907]	[0.000, 0.101, 0.899]
33	[0.057, 0.943, 0.000]	[0.000, 0.470, 0.530]	[0.024, 0.101, 0.876]	[0.000, 0.027, 0.973]
34	[0.183, 0.817, 0.000]	[0.045, 0.765, 0.190]	[0.005, 0.184, 0.810]	[0.000, 0.060, 0.940]
35	[0.676, 0.312, 0.012]	[0.183, 0.736, 0.082]	[0.033, 0.209, 0.758]	[0.000, 0.106, 0.894]
36	[0.232, 0.768, 0.000]	[0.188, 0.409, 0.403]	[0.000, 0.041, 0.959]	[0.000, 0.020, 0.980]
37	[0.050, 0.950, 0.000]	[0.002, 0.211, 0.787]	[0.003, 0.181, 0.816]	[0.014, 0.159, 0.828]
38	[0.139, 0.861, 0.000]	[0.260, 0.191, 0.549]	[0.000, 0.078, 0.922]	[0.000, 0.119, 0.881]
39	[0.931, 0.069, 0.000]	[0.011, 0.176, 0.813]	[0.000, 0.043, 0.957]	[0.000, 0.082, 0.918]
40	[**1.000, 0.000, 0.000**]	[0.012, 0.551, 0.436]	[0.000, 0.164, 0.836]	[0.005, 0.062, 0.933]
41	[0.032, 0.955, 0.013]	[0.081, 0.669, 0.250]	[0.007, 0.217, 0.776]	[0.003, 0.096, 0.901]
42	[0.148, 0.852, 0.000]	[0.051, 0.889, 0.060]	[0.000, 0.101, 0.899]	[0.000, 0.019, 0.981]
43	[0.094, 0.906, 0.000]	[0.061, 0.830, 0.109]	[0.000, 0.175, 0.825]	[0.010, 0.185, 0.805]
44	[0.743, 0.200, 0.057]	[0.014, 0.680, 0.306]	[0.000, 0.054, 0.946]	[0.000, 0.031, 0.969]
45	[0.253, 0.747, 0.000]	[0.008, 0.157, 0.835]	[0.000, 0.045, 0.955]	[0.000, 0.112, 0.888]
46	[0.755, 0.245, 0.000]	[0.061, 0.483, 0.456]	[0.037, 0.215, 0.748]	[0.000, 0.097, 0.903]
47	[0.000, 0.067, 0.933]	[0.180, 0.776, 0.044]	[0.000, 0.020, 0.980]	[0.000, 0.053, 0.947]
48	[0.876, 0.112, 0.012]	[0.024, 0.376, 0.600]	[0.003, 0.277, 0.720]	[0.000, 0.099, 0.901]
49	[0.215, 0.785, 0.000]	[0.122, 0.875, 0.003]	[0.000, 0.093, 0.907]	[0.000, 0.020, 0.980]
50	[0.134, 0.866, 0.000]	[0.254, 0.558, 0.188]	[0.000, 0.233, 0.767]	[0.000, 0.002, 0.998]

Table S4. The graphene dispersion state of [f_A, f_I, f_U] for graphene/p(MMA) nanocomposites with grafted chain lengths of n = 20, 50, and 70, respectively. f_A, f_I, and f_U represent the percentage of graphene beads in the dispersion state of Aggregated, Intercalated, and Unbound for each graphene.

n	$n = 3$	$n = 20$	$n = 50$	$n = 70$
Graphene ID	[f_A, f_I, f_U]	[f_A, f_I, f_U]	[f_A, f_I, f_U]	[f_A, f_I, f_U]
1	[0.049, 0.432, 0.519]	[0.041, 0.690, 0.270]	[0.046, 0.488, 0.466]	[0.001, 0.099, 0.900]
2	[0.061, 0.543, 0.396]	[0.124, 0.266, 0.611]	[0.104, 0.680, 0.216]	[0.024, 0.463, 0.514]
3	[0.128, 0.615, 0.257]	[0.108, 0.744, 0.147]	[0.000, 0.024, 0.976]	[0.026, 0.465, 0.509]
4	[0.104, 0.729, 0.166]	[0.100, 0.848, 0.051]	[0.035, 0.380, 0.585]	[0.000, 0.107, 0.893]
5	[0.188, 0.810, 0.002]	[0.158, 0.821, 0.020]	[0.245, 0.186, 0.569]	[0.000, 0.064, 0.936]
6	[0.334, 0.664, 0.003]	[0.077, 0.805, 0.118]	[0.089, 0.497, 0.414]	[0.000, 0.064, 0.936]
7	[0.233, 0.262, 0.505]	[0.096, 0.746, 0.158]	[0.005, 0.278, 0.716]	[0.000, 0.319, 0.681]
8	[0.071, 0.893, 0.036]	[0.142, 0.815, 0.044]	[0.035, 0.587, 0.378]	[0.000, 0.061, 0.939]
9	[0.324, 0.657, 0.019]	[0.045, 0.324, 0.630]	[0.000, 0.082, 0.918]	[0.000, 0.114, 0.886]
10	[0.295, 0.683, 0.022]	[0.036, 0.608, 0.355]	[0.010, 0.235, 0.755]	[0.000, 0.234, 0.766]
11	[0.481, 0.462, 0.057]	[0.005, 0.110, 0.884]	[0.008, 0.148, 0.844]	[0.005, 0.119, 0.876]
12	[0.272, 0.728, 0.000]	[0.066, 0.818, 0.117]	[0.003, 0.091, 0.906]	[0.000, 0.131, 0.869]
13	[0.439, 0.414, 0.146]	[0.280, 0.315, 0.405]	[0.084, 0.580, 0.335]	[0.117, 0.553, 0.330]
14	[0.000, 0.369, 0.631]	[0.212, 0.547, 0.240]	[0.365, 0.280, 0.355]	[0.105, 0.327, 0.568]
15	[0.071, 0.726, 0.204]	[0.250, 0.750, 0.000]	[0.006, 0.107, 0.887]	[0.000, 0.031, 0.969]
16	[0.496, 0.501, 0.003]	[0.104, 0.627, 0.269]	[0.008, 0.194, 0.798]	[0.000, 0.000, 1.000]
17	[0.038, 0.772, 0.190]	[0.006, 0.680, 0.314]	[0.014, 0.189, 0.797]	[0.000, 0.020, 0.980]
18	[0.207, 0.666, 0.127]	[0.244, 0.713, 0.042]	[0.053, 0.181, 0.766]	[0.000, 0.135, 0.865]
19	[0.112, 0.477, 0.411]	[0.146, 0.690, 0.164]	[0.315, 0.142, 0.543]	[0.000, 0.283, 0.717]
20	[0.181, 0.815, 0.004]	[0.340, 0.608, 0.051]	[0.008, 0.188, 0.803]	[0.026, 0.145, 0.829]
21	[0.330, 0.670, 0.000]	[0.069, 0.725, 0.206]	[0.003, 0.178, 0.818]	[0.000, 0.151, 0.849]
22	[0.303, 0.583, 0.113]	[0.000, 0.273, 0.727]	[0.013, 0.119, 0.868]	[0.000, 0.018, 0.982]
23	[0.149, 0.683, 0.168]	[0.085, 0.688, 0.228]	[0.063, 0.357, 0.580]	[0.000, 0.119, 0.881]
24	[0.352, 0.648, 0.001]	[0.252, 0.735, 0.012]	[0.054, 0.766, 0.180]	[0.003, 0.274, 0.724]
25	[0.071, 0.566, 0.364]	[0.199, 0.801, 0.000]	[0.128, 0.575, 0.296]	[0.000, 0.018, 0.982]
26	[0.166, 0.760, 0.074]	[0.054, 0.531, 0.415]	[0.081, 0.606, 0.313]	[0.027, 0.221, 0.752]
27	[0.145, 0.823, 0.032]	[0.226, 0.774, 0.000]	[0.252, 0.289, 0.458]	[0.000, 0.000, 1.000]
28	[0.263, 0.737, 0.000]	[0.040, 0.119, 0.840]	[0.111, 0.576, 0.313]	[0.000, 0.003, 0.997]
29	[0.190, 0.810, 0.000]	[0.128, 0.837, 0.035]	[0.185, 0.179, 0.636]	[0.102, 0.222, 0.676]
30	[0.139, 0.405, 0.456]	[0.096, 0.904, 0.000]	[0.000, 0.094, 0.906]	[0.007, 0.168, 0.825]
31	[0.260, 0.713, 0.027]	[0.018, 0.982, 0.000]	[0.070, 0.796, 0.134]	[0.000, 0.120, 0.880]
32	[0.114, 0.871, 0.015]	[0.076, 0.527, 0.398]	[0.000, 0.165, 0.835]	[0.113, 0.624, 0.263]
33	[0.359, 0.548, 0.093]	[0.091, 0.697, 0.212]	[0.042, 0.437, 0.521]	[0.000, 0.009, 0.991]
34	[0.280, 0.188, 0.532]	[0.084, 0.679, 0.236]	[0.129, 0.492, 0.379]	[0.000, 0.005, 0.995]
35	[0.154, 0.413, 0.433]	[0.072, 0.474, 0.454]	[0.000, 0.174, 0.826]	[0.000, 0.116, 0.884]
36	[0.179, 0.821, 0.000]	[0.085, 0.755, 0.159]	[0.011, 0.116, 0.873]	[0.000, 0.106, 0.894]
37	[0.256, 0.588, 0.156]	[0.098, 0.697, 0.206]	[0.000, 0.024, 0.976]	[0.000, 0.148, 0.852]
38	[0.283, 0.512, 0.204]	[0.075, 0.252, 0.673]	[0.070, 0.309, 0.620]	[0.000, 0.043, 0.957]
39	[0.150, 0.850, 0.000]	[0.074, 0.917, 0.009]	[0.027, 0.168, 0.805]	[0.000, 0.102, 0.898]
40	[0.095, 0.905, 0.000]	[0.080, 0.552, 0.368]	[0.064, 0.447, 0.489]	[0.000, 0.013, 0.987]
41	[0.296, 0.704, 0.000]	[0.050, 0.616, 0.334]	[0.241, 0.270, 0.489]	[0.000, 0.113, 0.887]
42	[0.130, 0.759, 0.111]	[0.192, 0.808, 0.000]	[0.278, 0.362, 0.360]	[0.000, 0.062, 0.938]
43	[0.267, 0.606, 0.127]	[0.084, 0.675, 0.241]	[0.023, 0.568, 0.409]	[0.000, 0.117, 0.883]
44	[0.183, 0.808, 0.009]	[0.079, 0.476, 0.445]	[0.019, 0.140, 0.841]	[0.000, 0.033, 0.967]
45	[0.031, 0.753, 0.216]	[0.094, 0.817, 0.089]	[0.101, 0.667, 0.232]	[0.004, 0.086, 0.910]
46	[0.236, 0.759, 0.005]	[0.167, 0.801, 0.032]	[0.186, 0.750, 0.064]	[0.003, 0.071, 0.926]
47	[0.159, 0.841, 0.000]	[0.090, 0.721, 0.189]	[0.161, 0.738, 0.101]	[0.021, 0.574, 0.404]
48	[0.184, 0.622, 0.194]	[0.074, 0.851, 0.076]	[0.070, 0.566, 0.364]	[0.005, 0.108, 0.888]
49	[0.146, 0.854, 0.000]	[0.082, 0.662, 0.256]	[0.103, 0.691, 0.206]	[0.008, 0.403, 0.589]
50	[0.024, 0.610, 0.365]	[0.199, 0.801, 0.000]	[0.090, 0.594, 0.316]	[0.000, 0.072, 0.928]

References

(1) Hsu, D. D.; Xia, W.; Arturo, S. G.; Keten, S. Systematic Method for Thermomechanically Consistent Coarse-Graining: A Universal Model for Methacrylate-Based Polymers. *J Chem. Theory Comput.* **2014**, *10*, 2514–2527.

(2) Ruiz, L.; Xia, W.; Meng, Z.; Keten, S. A coarse-grained model for the mechanical behavior of multi-layer graphene. *Carbon* **2015**, *82*, 103–115.

(3) Xia, W.; Ruiz, L.; Pugno, N. M.; Keten, S. Critical length scales and strain localization govern the mechanical performance of multi-layer graphene assemblies. *Nanoscale* **2016**, *8*, 6456–6462.

(4) Xia, W.; Vargas-Lara, F.; Keten, S.; Douglas, J. F. Structure and dynamics of a graphene melt. *ACS nano* **2018**, *12*, 5427–5435.

(5) Hansoge, N. K.; Huang, T.; Sinko, R.; Xia, W.; Chen, W.; Keten, S. Materials by design for stiff and tough hairy nanoparticle assemblies. *ACS nano* **2018**, *12*, 7946–7958.

(6) Wang, Y.; Meng, Z. Mechanical and viscoelastic properties of wrinkled graphene reinforced polymer nanocomposites-Effect of interlayer sliding within graphene sheets. *Carbon* **2021**, *177*, 128–137.

(7) Wang, Y.; Nie, W.; Wang, L.; Zhang, D.; Niu, K.; Xia, W. Understanding the graphene-polymer interfacial mechanical behavior via coarse-grained modeling. *Comput. Mater. Sci.* **2023**, *222*, 112109.

(8) Yang, Z.; Chiang, C.-C.; Meng, Z. Investigation of dynamic impact responses of layered polymer-graphene nanocomposite films using coarse-grained molecular dynamics simulations. *Carbon* **2023**, *203*, 202–210.

(9) Liao, Y.; Molares Palmero, O.; Arshad, A.; Chen, L.; Xia, W. Molecular Dynamics Simulations of Crumpling Polymer-Grafted Graphene Sheets: Implications for Functional Nanocomposites. *ACS Appl. Nano Mater.* **2024**, *7*, 7802–7811.

(10) Rissanou, A. N.; Harmandaris, V. Dynamics of various polymer-graphene interfacial systems through atomistic molecular dynamics simulations. *Soft Matter* **2014**, *10*, 2876–2888.

(11) Yuan, L.; Yao, X.; Yang, H. Multiscale modelling of strain-resistance behaviour for graphene rubber composites under large deformation. *Nanoscale* **2019**, *11*, 21554–21568.

(12) Wang, Y.; Li, Z.; Sun, D.; Jiang, N.; Niu, K.; Giuntoli, A.; Xia, W. Understanding the thermomechanical behavior of graphene-reinforced conjugated polymer nanocomposites via coarse-grained modeling. *Nanoscale* **2023**, *15*, 17124–17137.

(13) Rodríguez-Pérez, L.; Herranz, M. Á.; Martín, N. The chemistry of pristine graphene. *Chem. Commun.* **2013**, *49*, 3721–3735.

(14) Payne, M. C.; Teter, M. P.; Allan, D. C.; Arias, T. A.; Joannopoulos, J. D. Iterative Minimization Techniques for Abinitio Total-Energy Calculations - Molecular-Dynamics and Conjugate Gradients. *Rev. Mod. Phys.* **1992**, *64*, 1045–1097.

(15) Liu, Z.; Robinson, J. T.; Sun, X.; Dai, H. PEGylated nanographene oxide for delivery of water-insoluble cancer drugs. *J. Am. Chem. Soc.* **2008**, *130*, 10876–10877.

(16) Suter, J. L.; Sinclair, R. C.; Coveney, P. V. Principles governing control of aggregation and dispersion of graphene and graphene oxide in polymer melts. *Adv. Mater.* **2020**, *32*, 2003213.

(17) Wang, Y.; Li, Z.; Sun, D.; Jiang, N.; Niu, K.; Giuntoli, A.; Xia, W. Understanding the thermomechanical behavior of graphene-reinforced conjugated polymer nanocomposites via coarse-grained modeling. *Nanoscale* **2023**, *15*, 17124–17137.

(18) Muhammad, A.; Srivastava, R.; Koutroumanis, N.; Semitekolos, D.; Chiavazzo, E.; Pappas, P.-N.; Galiotis, C.; Asinari, P.; Charitidis, C. A.; Fasano, M. Mesoscopic modeling and experimental validation of thermal and mechanical properties of polypropylene nanocomposites reinforced by graphene-based fillers. *Macromolecules* **2023**, *56*, 9969–9982.

(19) Stukowski, A. Visualization and Analysis of Atomistic Simulation Data with OVITO-the Open Visualization Tool. *Modell. Simul. Mater. Sci. Eng.* **2010**, *18*, 015012.

(20) Krone, M.; Stone, J. E.; Ertl, T.; Schulten, K. Fast Visualization of Gaussian Density Surfaces for Molecular Dynamics and Particle System Trajectories. *EuroVis-Short Papers* **2012**, *10*, 67–71.

(21) Cha, J.; Kyoung, W.; Song, K.; Park, S.; Lim, T.; Lee, J.; Kang, H. Quantitative Evaluation of the Dispersion of Graphene Sheets With and Without Functional Groups Using Molecular Dynamics Simulations. *Nanoscale Res. Lett.* **2016**, *11*, 136.

(22) Payandehpeyman, J.; Mazaheri, M.; Khamehchi, M. Prediction of electrical conductivity of polymer-graphene nanocomposites by developing an analytical model considering interphase, tunneling and geometry effects. *Compos. Commun.* **2020**, *21*, 100364.
